# Minor differences in the untranslated regions of measles vector additional transcription units are reflected by differential immunogenicity of encoded MERS-CoV Spike antigen

**DOI:** 10.1128/jvi.00644-26

**Published:** 2026-07-01

**Authors:** Vishaka Tiwarekar, Aileen Ebenig, Yvonne Predota, Sabrina Schrauf, Cindy Hörner, Mona V. Lange, Mohamed R. Gadalla, Arne Auste, Csaba Miskey, Samada Muraleedharan, Constantine Konstantoulas, Christoph Schürmann, Daniela Lupinek, Anna H. Fiedler, Katrin Ramsauer, Erich Tauber, Michael D. Mühlebach

**Affiliations:** 1 Division of Veterinary Medicine, Paul-Ehrlich-Institut 39053 , Langen, Germany; 2 Themis Bioscience GmbH, acquired in 2020 by Merck & Co., Inc., Rahway, New Jersey, USA; 3 Division of Hematology, Cell and Gene Therapy, Paul-Ehrlich-Institut, Langen, Germany; University of Kentucky College of Medicine, Lexington, Kentucky, USA

**Keywords:** delivery vectors, heterologous antigen expression, platform technologies, emerging infections, measles, live vector vaccines, vaccines

## Abstract

The recombinant measles virus (MeV) is among those vaccine platform technologies, which are tested for the fast generation of effective vaccines against other pathogens, such as emerging virus diseases. Different live-attenuated vaccine strains have become available as backbones, with few differences. Here, we analyze how minor differences of recombinant Moraten (MV_vac2_) or Schwarz (MV_Schw_) constructs have an impact on the performance of respective derived Middle East respiratory syndrome (MERS) vaccine candidates. MV_vac2_ or MV_Schw_ viruses, both encoding the Spike glycoprotein (S) of the MERS-coronavirus (MERS-CoV) in an additional transcription unit (ATU) between the hemagglutinin gene and the polymerase gene L cassette, were tested. Both robustly expressed the additional gene. However, approximately twofold to threefold higher amounts of S proteins were observed in cells infected by the MV_Schw_-MERS-S(H) virus, correlating with the same relative ratio of S mRNA found in these cells. Exchange of the short genomic 3′ UTR ATU sequence in MV_vac2_ against the sequence in MV_Schw_ enhanced gene expression by about the same factor and demonstrates causality. MeV-susceptible IFNAR^−/−^-CD46Ge mice vaccinated with either candidate reveal that higher relative S antigen expression by MV_Schw_-MERS-S(H) tended toward induction of higher specific antibody titers, but lower numbers of S antigen-specific T cells. In subsequent challenge experiments, both candidates were effective. Thus, minor differences in the genetic composition of recombinant MeV’s ATU may modulate the gene expression pattern of additionally encoded proteins that are reflected by changes in the respective immunogenicities, but which were not decisive for protection in the chosen disease model.

**IMPORTANCE:**

In case of emerging or re-emerging infections, vaccine platform technologies are needed to rapidly develop effective vaccines to aid public healthcare in pandemics. Besides mRNA vaccines, also viral platform technologies, that is, the adenovirus-derived vaccines Vaxzevria and JCOVDEN, have proven to be of immense value during the COVID-19 pandemic. For future pandemics, it is crucial to understand the factors in vector design that modulate immunogenicity. This knowledge allows the tailoring of vaccine vectors to fit specific target product profiles, for example, to build vectors which trigger an accentuated T cell or, alternatively, antibody response against an antigen of interest. Our study using the live-attenuated measles vaccine backbone as a promising example is therefore crucial in demonstrating that very minor differences in the vaccine backbone can alter the antigen expression profile of the vector-antigen system and impact the relative induction of T-cell or antibody responses against the added target antigen

## INTRODUCTION

Measles is a highly contagious childhood disease that has caused millions of deaths in human history. The causative virus was first isolated in 1954, and by extended passaging on different cells in tissue culture, live-attenuated vaccine strains were generated. These vaccines are very effective with protection rates exceeding 85% after a single vaccination and are safe with only very low rates of adverse events (1). Measles vaccination is even recommended for HIV-infected patients as long as their CD4^+^ T-cell count is above a certain threshold that would indicate severe immunodeficiency. Measles vaccines are key components of routine vaccinations in children and save hundreds of thousands of lives each year, with the yearly death rate dropping from more than half a million deaths worldwide to an estimated 140,000 deaths in 2018 (2).

The measles virus (MeV) is the prototypic *Morbillivirus* and belongs, as a *Paramyxovirus,* to the order of *Mononegavirales*. Its genome consists of single-stranded RNA of approximately 16 kb in size in negative-sense orientation. Therefore, generation of genetically altered *Mononegavirales* from plasmid DNA is not trivial, since the genetic information of the (−)RNA genome cannot be utilized by eukaryotic cells with their own transcription/translation machinery. Instead, the viral polymerase L and its co-factors, the phosphoprotein P and the nucleoprotein N, have to be present in cells to assemble the viral ribonucleoprotein complex, which transcribes viral mRNA and replicates the RNA genome via a positive-sense antigenome intermediate. First developed for the rabies virus (3), rescue systems that provide the helper gene functions in addition to the viral genome *in trans* became also available for MeV (4). Early on, this technology was used to generate recombinant MeV that encodes additional proteins, that is, cytokines (5), marker proteins such as GFP (6), or antigens of other pathogens (7). Inclusion of additional heterologous genes into such recombinant viruses is quite straightforward due to the cassette-like organization of the MeV genome. The P gene cassette encodes, besides the P ORF, also the accessory proteins V and C. For all other viral proteins, each ORF is encoded in a single gene cassette, which is flanked by conserved intergenic regions that contain start and stop signals for the viral polymerase complex. By duplication of intergenic regions, additional transcription units (ATUs) are generated that can be utilized to express foreign ORFs (5). These additional ORFs can significantly expand the genome size of the recombinant MeV, with the successful generation of recombinant MeV that harbored more than an additional 5 kbp of genetic information (8). Despite this general flexibility, the nucleotide number of recombinant MeV has to be a multiple of six to give rise to a replicating virus (“rule-of-six” [9]).

The relative position of the ORF-containing ATU in the MeV genome can be used to modulate the expression of the additional protein due to the transcriptional gradient in the MeV genome from 3′ → 5′. Promoter-proximal genes are transcribed to a higher extent than distal genes near the 5′ end (10), because the MeV polymerase complex can only assemble in terminal promoter regions on the virus genome or antigenome. However, re-initiation of transcription at each intergenic region at the start signal of the 5′ gene is not 100% efficient. Then, the complex falls off the genome and cannot transcribe viral genes 5′ of that intergenic junction.

The option to additionally encode antigens of other pathogens allowed the generation of recombinant vaccine viruses that not only protect against measles but also induce immune responses against other pathogens. Thereby, recombinant MeV can be used and developed as a vector platform technology, which has been tested against a considerable range of mostly viral diseases (reviewed in reference 11). As a prominent example, MeV-derived vaccines against COVID-19 are under development (12–17). In the meantime, several different MeV strains have been tested as backbone to construct such recombinant vaccines: Edmonston-B (“NSe” [5]), Schwarz (18, 19), Moraten (20), AIK-C (21), Edmonston-Zagreb (22), HL (23), or HL_att_ (24). The majority of these (Edmonston-B, Schwarz, Moraten, and Edmonston-Zagreb) are derived from the same primary isolate and just differ in their passaging history (25). Therefore, the resulting recombinant vaccines have minor differences in the MeV vector backbone because of their genetic origin, but also some due to their cloning history. For example, Moraten and Schwarz vaccine strain MeV are identical on the nucleotide level and have been intensively used for generating recombinant vaccines (11), but slightly different cloning strategies were chosen to insert the additional transcription units (19, 20, 26). If and how such minor differences reflect on the performance of the derived recombinant vaccine has not yet been systematically analyzed.

Previously, a Moraten-strain-derived vaccine candidate additionally encoding the Spike glycoprotein of MERS-CoV, MV_vac2_-MERS-S(H), has already been studied. IFNAR^−/−^-CD46Ge mice immunized with MV_vac2_-MERS-S(H) developed binding and neutralizing antibodies (nAbs) already after a single immunization, and nAb titers were boosted fivefold to sevenfold by the second vaccination (27). Furthermore, MV_vac2_-MERS-S(H) induced considerable amounts of CD8^+^ T cells expressing interferon-gamma (IFN-γ) or tumor necrosis factor alpha (TNF-α) upon re-stimulation with the immunodominant peptide of MERS-S, 75% of those reacting with both cytokines (28). Therefore, this well-described vaccine model offered the excellent opportunity to analyze to what degree a switch of the backbone strain will impact the vaccine’s efficacy.

Here, we directly compared Moraten- and Schwarz-strain-derived recombinant vaccine viruses that additionally encode codon-optimized, full-length Spike glycoprotein S of the Middle East respiratory syndrome coronavirus (MERS-CoV) from ATUs in the same genomic position, that is, following the hemagglutinin gene cassette. We demonstrated the vaccine strain-like replication of both candidates and analyzed the expression of the inserted gene by quantification of the relative abundance of mRNAs and antigens. Differences in gene expression were demonstrated to result from the flanking sequence of the additional transcription units, and the impact of this differential antigen expression was analyzed in appropriate transgenic mouse models for immunogenicity and protection against MERS-CoV-induced fulminant disease.

## MATERIALS AND METHODS

### Cells

Vero (African green monkey kidney) (ATCC# CCL-81) and 293T (ATCC CRL-3216) cell lines were purchased from ATCC (Manassas, VA, USA) and cultured in Dulbecco’s modified Eagle’s medium (DMEM, Biowest, Nuaillé, France) supplemented with 10% fetal bovine serum (FBS; Biochrom, Berlin, Germany) and 2 mM L-glutamine (L-Gln; Biochrom). All cells were cultured at 37°C in a humidified atmosphere containing 6% CO_2_ for a maximum of 6 months of culture after thawing of the original stock.

### Plasmids

pBRPolIIΔ-MV_vac2_-GFP(H), pCA-MV-N, pCA-MV-P, and pCA-MV-L have been described before (12). The *gfp* gene in the Schwarz-strain ATU, but otherwise flanked by Moraten-strain sequences between *MluI* and *AflII* restriction sites in plasmid pMK-SchwGFPH, was obtained by gene synthesis (Invitrogen Life Technology, Regensburg, Germany). pBRPolII-MeV_vac2_-SchwGFP(H) was constructed by first generating the cloning intermediate pBRMV_vac2_-GFP/L via cutting pBRPolIIΔ-MV_vac2_-GFP(H) with *AscI/KlfI* and re-ligating the blunted plasmid backbone. The GFP-ORF in a Schwarz-strain ATU and surrounding Moraten sequences between *MluI* and *AflII* sites was cut out from pMK-SchwGFPH using these restriction endonucleases and ligated into pBRMV_vac2_-GFP/L opened via the same enzymes to yield pBRMV_vac2_-SchwGFP-L. This plasmid gave rise to pBRPolIIΔ-MV_vac2_-GFP(SchwH) after opening with *MluI* and *SfiI* and insertion of the *MluI/SfiI*-fragment of pBRPolIIΔ-MV_vac2_-GFP(H) encoding the rest of the MeV Moraten-strain genome. To exchange the *gfp* gene against the MERS-CoV Spike protein gene in this backbone, the S gene optimized for human codon usage was amplified from pMA-RQ-MERS-S (27) using the following primers: Forward 5′-AACGCGTACGATGATCCACTCCGTGTTTCTGCTGA-3′, reverse 5′- CGAGACGTCTAAGCGCTGCGCGCTCAGTGCACATGCACTTTGTGAGGTTCCAGGTC-3′. The amplicon was cloned into pCR2.1-Topo (Fisher Scientific GmbH, Schwerte, Germany). After sequencing, the gene was sub-cloned into pBRPolIIΔ-MV_vac2_-GFP(SchwH) instead of the GFP cassette via *MluI* and *AatII* restriction sites to generate pBRPolIIΔ-MV_vac2_-MERS-S(SchwH).

To generate vaccine virus genomes with hybrid ATUs, the MERS-CoV S gene was amplified from pMA-RQ-MERS-S (27) using the primers as defined in Table S1. The amplicons were cloned into pCR2.1-Topo (Fisher Scientific GmbH) to yield pCR2.1-Moraten-MERS-CoV-S-Schwarz and pCR2.1-Schwarz-MERS-CoV-S-Moraten. After sequencing, the gene cassettes were sub-cloned into pBRPolIIΔ-MV_vac2_-GFP(SchwH) or pBRPolIIΔ-MV_vac2_-GFP(H) via *MluI* and *AatII* restriction sites to generate pBRPolIIΔ-MV_vac2_-MERS-S(Mor/Schw H) or pBRPolIIΔ-MV_vac2_-MERS-S(Schw/Mor H), respectively.

pTM-MVSchw, which contains an infectious MeV cDNA corresponding to the antigenome of the Schwarz MeV vaccine strain, and pEMC-La, which expresses the MeV polymerase L gene, have been described elsewhere (19). To generate pTM-MV_Schw_-MERS vectors, a cDNA encoding the full-length Spike glycoprotein S of MERS-CoV was obtained by gene synthesis (GenScript, USA). The sequence was codon optimized for expression in mammalian cells, and MV editing-like sequences were mutated. The complete sequence respects the “rule of six” and contains BsiWI and BssHII restriction sites at either end, respectively. This cDNA was inserted into BsiWI/BssHII-digested pTM-MVSchw-ATU3, which contains an ATU between the hemagglutinin (H) and polymerase (L) gene cassettes of the Schwarz MV genome.

### Viruses

Chimeric MeV_vac2_-GFP(SchwH), MeV_vac2_-MERS-S(SchwH), MeV_vac2_-MERS-S(Mor/Schw H), and MeV_vac2_-MERS-S(Schw/Mor H) viruses were rescued as described (29). In brief, 5 μg of MeV genome plasmids pBRPolIΔ-MV_vac2_-SchwGFP(H), pBRPolIΔ-MV_vac2_-MERS-S(SchwH), pBRPolIIΔ-MV_vac2_-MERS-S(Mor/Schw H), or pBRPolIIΔ-MV_vac2_-MERS-S(Schw/Mor H) was co-transfected with plasmids pCA-MV-N (0.4 μg), pCA-MV-P (0.1 μg), and pCA-MV-L (0.4 μg) encoding MeV proteins necessary for genome replication and expression in 293T cells cultured in six-well plates using Lipofectamine 2000 (Invitrogen Life Technology). The transfected 293T cells were overlaid 2 days after transfection onto 50% confluent Vero cells seeded in 10 cm dishes. Overlay cultures were closely monitored for isolated syncytia, indicating monoclonal replicative centers. Single syncytia were picked and overlaid onto 50% confluent Vero cells cultured in six-well plates and harvested as “passage 0” (P0) by scraping and a freeze-thaw cycle of cells at the time of maximal infection.

Rescue of recombinant MV_Schw_-MERS-S(H) from pTM-MVSchw-MERS plasmid was performed as previously described (19) using the helper-cell-based rescue system described by Radecke et al. (4) and modified by Parks et al. (30). Briefly, 293 T7/N/P cells, stably expressing the T7 polymerase and the MeV N and P proteins, were transfected with pTM-MVSchw (3 μg) and pEMC-La (12 ng), using JetPEI (Polyplus, Illkirch, France) according to the manufacturer’s instructions. After overnight incubation at 37°C, the transfection medium was replaced by fresh medium, and the cells were heat shocked at 43°C for 3 h and then returned to 37°C. After 2 days of incubation at 37°C, transfected cells were transferred onto Vero cells and incubated at 37°C. Single syncytia were picked and overlaid onto 80% confluent Vero cells cultured in 12-well plates and harvested as “passage 0” (P0) by scraping and a freeze-thaw cycle of cells at the time of maximal infection.

MERS-S encoding vaccine virus MV_vac2_-MERS-S(H) (27), MV_Schw_-MERS-S(H), and control viruses MV_vac2_-ATU(P) (27) or MV_Schw_ (19) were used in P4 for vaccination. These as well as MV_vac2_-GFP(P), MeV_vac2_-GFP(SchwH), MeV_vac2_-MERS-S(SchwH), MeV_vac2_-MERS-S(Mor/Schw H), MeV_vac2_-MERS-S(Schw/Mor H), and MERS-CoV (isolate EMC/2012) (31) were propagated and titrated on Vero cells by the TCID_50_ limited dilution method of Spearman and Kaerber (32, 33). All virus stocks were stored in aliquots at −80°C. Multistep viral growth kinetics were analyzed by infecting Vero cells at an MOI of 0.03 in 12-well plates and incubating at 37°C. At various time points, supernatants were clarified by centrifugation, and cells were scraped into OptiMEM and subjected to freeze-thaw cycles. Released and cell-associated viral titers were determined by titration of the TCID_50_.

### High-throughput virus genome sequencing and read processing

Virus RNA was isolated from supernatant of Vero cell cultures using the Quick RNA Viral Kit (Cat. No. R1034, Zymo Research, Freiburg, Germany) after benzonase treatment of the supernatant to digest all non-encapsulated nucleic acids, as follows. Two microliters of benzonase (Merck Millipore) and 80 μL of 25 mM MgCl_2_ were added to 920 μL supernatant of infected cell culture (Vero cells infected at an MOI of 0.01) and incubated at 37°C for 30 min, and 0.1 M EDTA was used to stop the reaction. Subsequently, proteinase K (5 μg/mL; Invitrogen) was added, and the mixture was incubated at 37°C for 1 h.

The isolated virus RNA was reverse transcribed using Maxima H Minus Reverse Transcriptase (Thermo Fisher) and random hexamers for 1 h in a 20 μL reaction volume. Second-strand synthesis was done with the NEBNext Ultra II Non-Directional RNA Second-Strand Synthesis Module (NEB). After magnetic bead purification, the cDNA was fragmented with the NEBNext Ultra II FS DNA Module and ligated with 1.25 pmol Illumina adapters, using the NEBNext Ultra II Ligation Module. The magnetic-bead-purified DNA was amplified and barcoded by PCR using 5–9 cycles after determining the required minimum cycle numbers by qPCR, performed on one-tenth of the total ligated DNA amounts. The PCR products were gel isolated and sequenced on the NextSeq 2000 instrument (Illumina), with 2 × 66 paired-end setting.

The raw reads were quality-, adaptor-, and minimum length-trimmed with fastp (34). The reads were mapped against the Vero genome, and the non-mapping reads were used to assemble the virus genomes in a non-referenced-based manner using MEGAHIT (35) with the following settings: --k-step 10, --k-min 31, --min-count 4, and -no-mercy. To obtain sequence variation frequencies, the Vero-filtered reads were remapped to the nearly full-length genomic contigs (from MEGAHIT) using bowtie2 (36). Variants were called with FreeBayes (https://arxiv.org/abs/1207.3907) with the settings: -C 1, --pooled-continuous. Coverage was calculated from the deduplicated bam output files (of bowtie2) using BEDTools (37) and plotted in the R environment (https://www.R-project.org).

### Immunoperoxidase monolayer assay

For the immunoperoxidase monolayer assay, Vero cells cultured in flat-bottom 12-well plates were fixed overnight with methanol at −20°C 2 days after infection with an MOI of 0.01. The fixed cells were then washed three times with 1 mL PBS and subsequently blocked with PBS containing 2% bovine serum albumin (BSA) (Roth, Karlsruhe, Germany) for 30 min at 37°C. The cells were then probed for 1 h with a monoclonal chimeric anti-MERS-S protein antibody clone D12 (1:1,000; Ab00696-23.0; Absolute Antibody, Redcar, UK) or a rabbit anti-MeV N protein antibody (1:1,000, ab23974, Abcam) in PBS with 2% BSA. The cells were washed three times with 1 mL PBS and subsequently incubated with the secondary HRP-coupled donkey anti-rabbit IgG (H + L) polyclonal antibody (1:1,000; 611-7202; Rockland, Gilbertsville, USA) for 1 h at 37°C. Then, the cells were washed three times again. For detection, the cells were stained with TrueBlue peroxidase substrate solution (SeraCare, Milford, USA).

### Western blot analysis

Cells were lysed and immunoblotted as previously described (38). Mouse anti-MERS-S2 protein antibody (1:1,000; 40070-MM11; Sino Biological, Eschborn, Germany) and rabbit anti-MeV-N protein polyclonal antibody (1:5,000; ab23974; Abcam) were used. Donkey anti-mouse-HRP (1:10,000; AP192P; Sigma-Aldrich, Darmstadt, Germany) or anti-rabbit IgG-HRP (H&L) polyclonal antibody (1:10,000; 611-7202; Rockland) served as secondary antibody. Peroxidase activity was visualized with an enhanced chemiluminescence detection kit (Thermo Scientific, Bremen, Germany) on ChemiDoc MP Imaging System (Biorad, Dreieich, Germany).

### Quantitative reverse-transcription PCR

Viral mRNAs coding for MeV-N, MeV-H, MERS-CoV-S, or the cellular housekeeping protein GAPDH were quantified via quantitative reverse transcription-PCR (qRT-PCR) using Superscript II RT (Invitrogen, Darmstadt, Germany) and the LightCycler SYBR Green I Master kit (Roche, Basel, Switzerland) using a LightCycler II thermocycler (Roche). cDNA of different genes was reverse transcribed using Oligo(dT)12–18 primers and SuperScript II RT with 2 μg RNA, 1 mM dNTPs, 0.1 mM DTT in a total volume of 20 μL. First-strand buffer was used by incubation for 50 min at 42°C after initial denaturation of RNA for 5 min at 65°C. cDNA synthesis was stopped by inactivating the reverse transcriptase for 15 min at 70°C. Two microliters of cDNA was used per reaction as the template for subsequent analysis by qPCR. Alternatively, mRNAs of the respective samples were quantified by one-step qPCR using QuantiTect SYBR Green RT-PCR Kit (Qiagen) and LightCycler480 II (Roche) in a total reaction volume of 10 μL. Primers for qPCR amplification are listed in Table S2. For analysis of samples, plasmids pCA-MV-N (29), pCG-H (39), or pMA-RQ-MERS-solS (27) of known concentration were serially diluted and applied as DNA standards for copy number calculation.

The cycling conditions were as follows: Denaturation for 10 s at 95°C, and 45 cycles of 15 s at 95°C, 10 s at 58°C, and 15 s at 72°C, when the fluorescent signal was acquired. After 45 cycles, melting curves of amplified products were recorded before cooling to 40°C.

### DI RNA detection

DI-RNA analysis was performed as previously described (40). In brief, Vero cells were infected with viruses of interest with an MOI of 0.1. Cells were incubated at 37°C for 48 h prior to total RNA isolation using TRIzol reagent (Ambion, Thermo Fisher Scientific). cDNA was generated from 1 µg of total RNA using SuperScript II (Invitrogen Life Technologies) and random hexamer (Invitrogen Life Technologies) in a total volume of 20 μL. One microliter of the resulting cDNA was amplified using two DI-specific primers (A1 and A2 [40, 41]) and Taq polymerase (New England Biolabs) in a total volume of 50 µL. The following PCR conditions were used: Initial denaturation at 95°C for 30 s, followed by 30 cycles of denaturation (95°C, 30 s), annealing (48°C, 30 s), and elongation (68°C, 1 min). Final elongation at 68°C for 5 min followed. The generated products were analyzed using a 2%TAE agarose gel and 1 kb Plus DNA Ladder (New England Biolabs). Additionally, a control PCR for full-length RNA was performed using a combination of previously published primers A2 and B1 (41).

### Animal experiments

IFNAR^−/−^-hCD46Ge^+/−^ mice were bred by crossing IFNAR^−/−^-CD46Ge^+/−^ animals with IFNAR^−/−^ mice (42, 43) and checking for the presence of the CD46Ge transgene in offspring by PCR genotyping as described (44). IFNAR^−/−^-hDPP4^+/−^ mice were bred by crossing the hDPP4 transgene from line 52 hCD26 Tg^+^ mice (kind gift of K. Tseng, UTMB) (45) into the IFNAR^−/−^ mice. Mice were bred for homozygosity of the IFNARko allele, while the hDPP4 was heterozygously maintained by breeding IFNAR^−/−^-hDPP4^+/−^ animals with IFNAR^−/−^ mice and checking for the presence of the DPP4 transgene in offspring by PCR genotyping as described (45).

For the immunization experiments, animals were randomized for age- and sex-matched groups and housed in IVC cages in groups of 3–5 animals with nist packs as environmental enrichment at room temperature with regular 12-h day and 12-h night intervals. Group sizes were calculated based on statistical considerations to yield sufficient statistical power as authorized by the respective competent authority. These animals were inoculated intraperitoneally (i.p.) with 1 × 10^5^ TCID_50_ of recombinant vaccine viruses in 200 μL volume. In total, 200 μL blood was collected on days 0 and 28 by submandibular bleeding, while final serum for analysis of humoral responses was collected by heart bleed under ketamine-xylazine anesthesia on day 49 post-initial immunization (p.i.). Serum samples were stored at −20°C. For analysis of cellular immune responses, mice were prime-boost immunized as described, but euthanized on day 35 p.i., and splenocytes were harvested for assessment of cellular immune responses.

For challenge experiments, 6- to 12-week-old IFNAR^−/−^-hDPP4^+/−^ mice were vaccinated on days 0 and 28 as described above. Blood was drawn on days 0, 28, and on the day of challenge. On day 49 p.i., mice were challenged by applying the indicated doses of MERS-CoV EMC/2012 intranasally (i.n.) in a volume of 30 μL.

### IFN-γ ELISpot analysis

Murine IFN-γ enzyme-linked immunosorbent spot (ELISpot) assays were performed as described (28), using the Mouse IFN-γ ELISpot Pair kit, including capture and detection antibody (BD Biosciences, Franklin Lakes, NJ, USA) and HRP Streptavidin (BD Biosciences) for ELISpot detection in combination with multiscreen immunoprecipitation (IP) ELISpot polyvinylidene difluoride (PVDF) 96-well plates (Merck Millipore, Darmstadt, Germany) according to the manufacturer’s instructions. 5 × 10^5^ isolated splenocytes were cultured with different stimuli in 200 μL RPMI +10% FBS, 2 mM L-Gln, and 1% penicillin-streptomycin for 36 h. For re-stimulation of MERS-CoV S protein-specific T cells, isolated splenocytes were cultivated in the presence of 10 μg/mL MERS-CoV S-derived peptide S1165 (Biosynthesis Inc., Lewisville, TX, USA [46]) or 10 μg/mL SIINFEKL control peptide (SIN) of ovalbumin (aa 257–264) (InvivoGen, San Diego, CA, USA), as appropriate. In parallel, splenocytes were stimulated with 10 μg/mL MeV bulk antigen (Virion Serion, Würzburg, Germany). For general T-cell stimulation, 10 μg/mL concanavalin A (ConA, Sigma-Aldrich) was used, and as a negative control, splenocytes were left untreated. After 36 h, cells were spun down, supernatants were removed, and cells were lysed in the wells by hypotonic shock. Plates were incubated with biotin-conjugated anti-IFN-γ detection antibodies and streptavidin-HRP according to the manufacturer’s instructions. 3-Amino-9-ethyl-carbazole (AEC; Sigma-Aldrich) was dissolved in N,N-dimethylformamide (Merck Millipore) and used for peroxidase-dependent staining. Spots were counted using an Eli.Scan ELISpot scanner (AE.L.VIS, Hamburg, Germany) and ELISpot analysis software Eli.Analyse V5.0 (AE.L.VIS).

### Intracellular cytokine staining

For flow cytometry-based analysis of cytokine expression by intracellular cytokine staining (ICS), splenocytes of vaccinated mice were treated and analyzed as described (12). In short, splenocytes were stimulated for 5 h and stained for cell surface markers CD3, CD4, and CD8, as well as for cytokines IFN-γ, IL-2, and TNF-α. Fixed cells were analyzed via flow cytometry using an LSRII SORP flow cytometer (BD Biosciences) and DIVA software (BD Biosciences).

### ELISA

MeV bulk antigens (10 μg/mL; Virion Serion) or recombinant MERS-CoV S protein (20 μg/mL) were coated in 50 μL carbonate buffer (Na_2_CO_3_ 30 mM; NaHCO_3_ 70 mM; pH 9.6) per well on Nunc Maxisorp 96-well ELISA plates (ebioscience) and incubated overnight at 4°C. The plates were washed three times with 200 μL ELISA washing buffer (PBS, 0.1% Tween 20 [wt/vol]) and blocked with 100 μL blocking buffer (PBS; 5% BSA; 0.1% Tween 20) for at least 2 h at room temperature. Mouse sera were fivefold serially diluted in ELISA dilution buffer (PBS, 1% BSA, 0.1% Tween 20), and 50 μL/well was used for the assay. The plates were incubated at 37°C for 2 h and washed three times with ELISA washing buffer, followed by incubation with 50 μL/well of HRP-conjugated rabbit anti-mouse total IgG (1:1,000 in ELISA dilution buffer; P0260, Dako Agilent, Santa Clara, CA, USA) at room temperature for 1 h. Subsequently, the plates were washed four times, and 100 μL TMB substrate (ebioscience) was added per well. The reaction was stopped by the addition of 50 μL/well H_2_SO_4_ (1 N), and the absorbance at 450 nm (specific signal) and 630 nm (reference wavelength) was measured.

### Virus neutralization test

Virus neutralizing titers (VNTs) were quantified as described previously (27). In brief, mouse sera were serially diluted in twofold dilution steps in DMEM in duplicates. A total of 50 PFU of MV_vac2_-GFP(P) or 200 TCID_50_ of MERS-CoV (isolate EMC/2012) was mixed with diluted sera and incubated at 37°C for 1 h. MeV or MERS-CoV virus-serum suspensions were added to 1 × 10^4^ Vero or Vero E6 cells, respectively, seeded 4 h prior to the assay in 96-well plates, and incubated for 4 days at 37°C. VNTs were calculated as the reciprocal of the highest mean dilution that abolished infection.

## RESULTS

### Generation of Schwarz-derived MERS vaccine candidate

To generate a comparator vaccine candidate for the Moraten-strain-derived MV_vac2_-MERS-S(H) (27, 28) based on a Schwarz backbone, the same codon-optimized cDNA encoding for the full-length Spike glycoprotein S of MERS-CoV was inserted into an additional transcription unit within the Schwarz MeV vector (19), between the hemagglutinin (H) and polymerase (L) gene cassettes. The recombinant MV_Schw_-MERS-S(H) virus was rescued after transfecting this plasmid into human helper cells and subsequent propagation on Vero cells.

**Fig 1:**
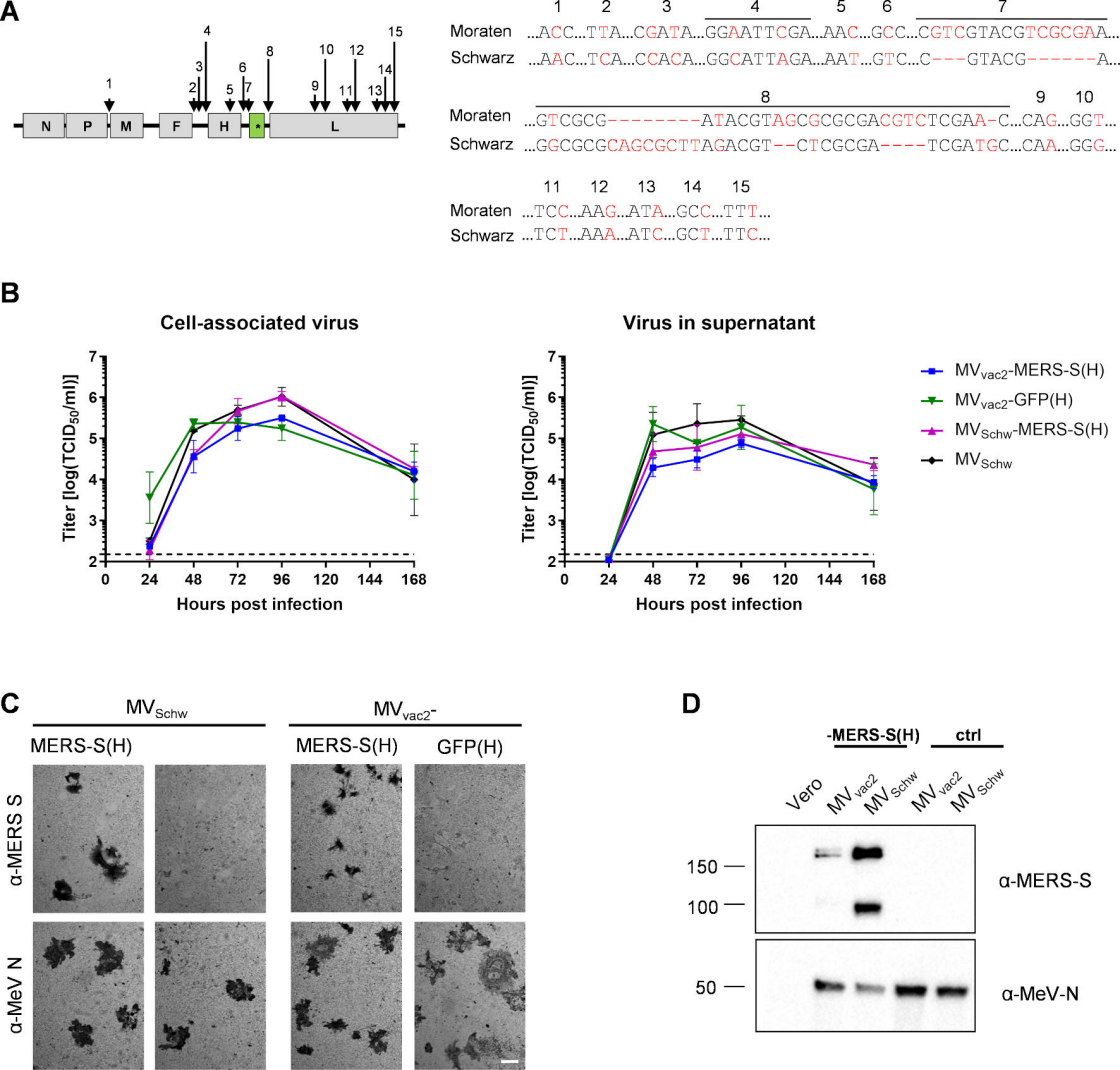
Genetic differences and *in vitro* characterization of Moraten- and Schwarz-strain-derived MERS vaccines MV_vac2_-MERS-S(H) and MV_Schw_-MERS-S(H). (**A**) Schematic depiction of MeV genome (left) with an ATU following the H gene marked in green and by an asterisk, indicating the 17 positions of differences between the parental recombinant Moraten and Schwarz genomes, respectively. These positions are detailed on the nucleotide level (right) with corresponding numbers and different nucleotides marked in red font, with positions 7 and 8 flanking the ATU. (**B**) Growth kinetics of recombinant MeV on Vero B4 cells infected at an MOI of 0.03 with MV_vac2_-MERS-S(H) and MV_Schw_-MERS-S(H) encoding MERS-CoV S in the post H position or control viruses (ctrl) MV_vac2_-GFP(H) or MV_Schw_. Titers of samples prepared at the indicated time points post-infection were titrated on Vero B4 cells. Means and standard deviations of three independent experiments are presented. (**C**) MERS-CoV S protein expression in Vero cells was verified via the immunoperoxidase monolayer assay. Scale bar represents 500 μm. (**D**) Immunoblot analysis of Vero B4 cells infected at an MOI of 0.01 with MV_vac2_-MERS-S(H), MV_vac2_-GFP(H), MV_Schw_-MERS-S(H), or MV_Schw_ as depicted above lanes. Uninfected cells (Vero) served as mock. Blots were probed using anti-MERS-CoV Spike antibody (upper blot) or mAb reactive against MeV-N (lower blot).

Unmodified Moraten and Schwarz strain Measles vaccine viruses are sequence identical. Therefore, only due to different cloning strategies of recombinant vaccine vector backbones, the alignment of both recombinant MERS vaccines revealed rather minor differences. Thereby, 17 non-coding or conserved point mutations are found, all but one in the 5′ halves of the genome, in addition to the region of the genome defining the ATU between the H and the L gene cassettes (Fig. 1A, Table 1).

**TABLE 1:** Nucleotide differences between genomes of recombinant Moraten- and Schwarz-strain-derived viruses^*a*^

Nucleotide position Moraten	Sequence Moraten	Nucleotide position Schwarz	Sequence Schwarz	AA exchange	Location
3373	A***C***C	3373	A***A***C	n.a.	UTR between N and P
7243	T***T***A	7243	T***C***A	n.a.	UTR between F and H
7267–7269	C***G***A***T***A	7267–7269	C***C***A***C***A	n.a.	UTR between F and H
7441–7445	GG***A***ATT***C***GA	7441–7445	GG***C***ATT***A***GA	No	H ORF
8353	AA***C***C	8353	AA***T***C	No	H ORF
9180	G***C***C	9180	G***T***C	n.a.	UTR between H and L prior to inserted ATU
9246–9260	C***GTC***GTACG***TCGCGA***A	9246–9252	C***---***GTACG***------***A	n.a.	ATU site between H and L, UTR
9981–10009	G***T***CGCG***--------***A***T***ACGT***AG***C***G***CG CGA***CGTC***TCGA***A******-***C	9972–10003	G***G***CGCG***CAGCGCTT*** A***G***ACGT***—***C***T***CG CGA***----***TCGA***TG***C	n.a.	ATU site between H and L, UTR
13007	CA***G***G	13001	CA***A***G	No	L ORF
13349	GG***T***T	13343	GG***G***T	No	L ORF
15077	TC***C***C	15071	TC***T***C	No	L ORF
15464	AA***G***G	15458	AA***A***G	No	L ORF
15920	AT***A***T	15914	AT***C***T	No	L ORF
16316	GC***C***T	16310	GC***T***T	No	L ORF
16376	TT***T***T	16370	TT***C***T	No	L ORF

^*a*^ Sequence alignment of MV_vac2_-GFP(H) (GenBank accession no. MH144178.1) and MV_schw_-GFP(ATU3) (GenBank accession no. LP940813.1). Variable nucleotides are indicated in boldface italic type; affected codons are depicted by underlining. UTR, untranslated region; ORF, open reading frame; ATU, additional transcription unit; n.a., not applicable.

### Determination of *in vitro* properties of recombinant MERS vaccines

Multi-step growth kinetics on the recombinant viruses and parental measles strains were performed to assess the potential inhibitory effects of the expressed MERS-CoV S transgene on recombinant virus replication by infection of Vero cells at an MOI of 0.03. Cell-associated virus and virus titers in supernatants of infected cells were titrated and revealed comparable growth of all recombinant viruses (Fig. 1B). Cell-associated virus titers reached a maximum of about 1 × 10^6^ TCID_50_/mL after 4 days of infection, while titers in the supernatant were slightly lower, peaking at around 3 × 10^5^ TCID_50_/mL. To check for uniform expression of the additionally encoded S antigen, Vero cell monolayers infected at low MOI were fixed and stained for expression of MERS-CoV S (Fig. 1C, upper panel) or MeV-N as control (Fig. 1C, lower panel). All virus-induced syncytia were stained for MERS-CoV S specifically for the cultures infected with MV_Schw_-MERS-S(H) or MV_vac2_-MERS-S(H), thereby revealing homogeneity of the vaccine virus quasi-species, also confirmed by sequencing the complete vRNA genome of MV_vac2_-MERS-S(H) after five passages (Fig. S1). To assess the quantity of expressed S-antigen, lysates of infected Vero cells (MOI = 0.1) were checked by Western blot analysis for expression of MERS-CoV S, and MeV N (Fig. 1D). Lanes loaded with lysates of both cultures infected with MV_Schw_-MERS-S(H) or MV_vac2_-MERS-S(H) stained positive for MERS-CoV S at the expected molecular weight. Surprisingly, cells infected with MV_Schw_-MERS-S(H) reproducibly expressed considerably more MERS-CoV S (3- to 10-fold more as assessed by exposure times) than cells infected by MV_vac2_-MERS-S(H), despite the genetic stability of the S-encoding gene cassette over at least 10 passages of MV_vac2_-MERS-S(H).

### Determining the mechanism of differential antigen expression

**Fig 2:**
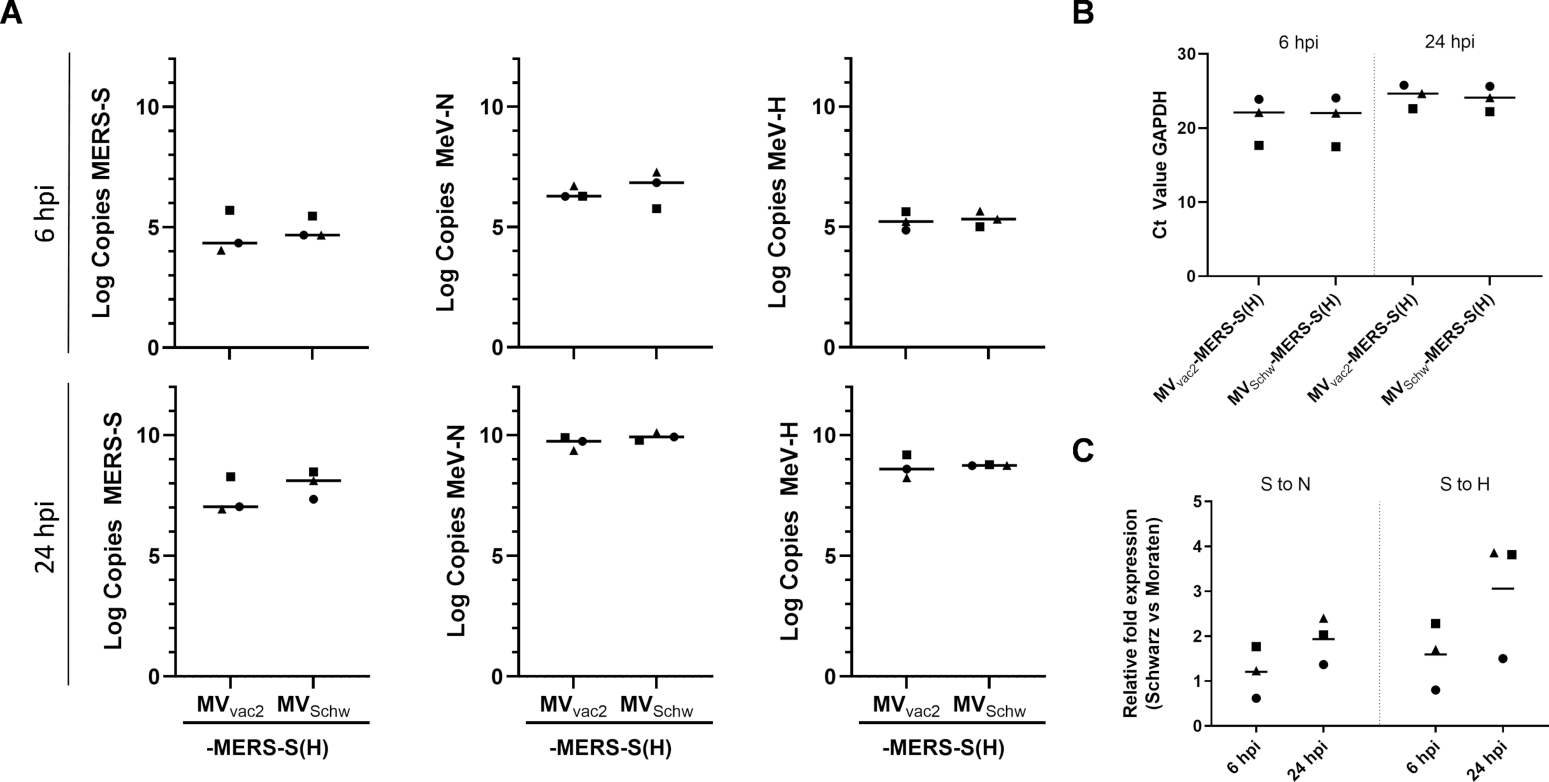
RNA abundance in cells replicating Moraten- or Schwarz-strain MERS vaccines. Abundance of vaccine (MeV-N, MeV-H) and antigen (MERS-S) mRNA during early (6 hpi) and later (24 hpi) stages of infection. (**A**) Absolute mRNA copy numbers of vaccine-encoded genes were determined by quantitative RT-PCR using a plasmid standard for each ORF. (**B**) Similar ct values of GAPDH mRNA in Vero cells infected with either MV_vac2_-MERS-S(H) or MV_schw_-MERS-S(H) demonstrate comparable numbers of cells in the tested samples of infected cultures. (**C**) Relative fold change expression of MERS-CoV S mRNA was determined by employing the ΔΔct method on qPCR data. mRNA encoding MeV N (left) or MeV H (right) were used as a gene for normalization. Samples from MV_vac2_-MERS-S(H) served as reference. Single dots represent results of independent experiments. Triangle: first experiment; circle: second experiment; square: third experiment.

To determine the mechanism causative for this considerably different protein expression of the additional S antigen between these highly similar recombinant viruses differing by only approximately 3‰ of their nucleotide sequence, we assessed the abundance of single mRNAs in infected cells early (6 h) and late (24 h) after infection by qRT-PCR using poly-dT primers for generating cDNA. mRNAs coding for MERS-CoV S as well as those coding for MeV-N of MeV-H were quantified (Fig. 2A). The relative abundance of S vs. N or S vs. H was calculated for each virus, and the fold difference in the relative abundance in Schwarz- vs. Moraten-infected cells is shown for each time point (Fig. 2C).

While early after infection, relative S mRNA abundance was comparable, S mRNA became 1.5- to 3-fold more abundant in cells infected with the Schwarz vaccine during late time points after infection (Fig. 2C). The comparable abundance early after infection argues against a difference in the expression strength of S mRNA, which should anyway mainly be determined by the transcriptional gradient of the MeV genome given the similar replication kinetics of both Moraten- and Schwarz-derived vaccine candidates. Enrichment of S mRNA in the Schwarz-infected cells over time instead hints at differences in mRNA stability, which may be determined by untranslated regions flanking the ORFs. Moreover, the alignment of recombinant vaccine virus genomes (Fig. 1A; Fig. S2) had already revealed the ATU region as a hotspot of difference between the Schwarz- and Moraten-strain-derived MERS vaccines. Therefore, we generated a recombinant MeV consisting of the Moraten-strain backbone using the identical Spike ORF again as in MV_vac2_-MERS-S(H), but encompassing the complete ATU sequence of the Schwarz clone used in this study. This strategy resulted in the recombinant virus MeV_vac2_-MERS-S(SchwH) (Fig. S2).

**Fig 3:**
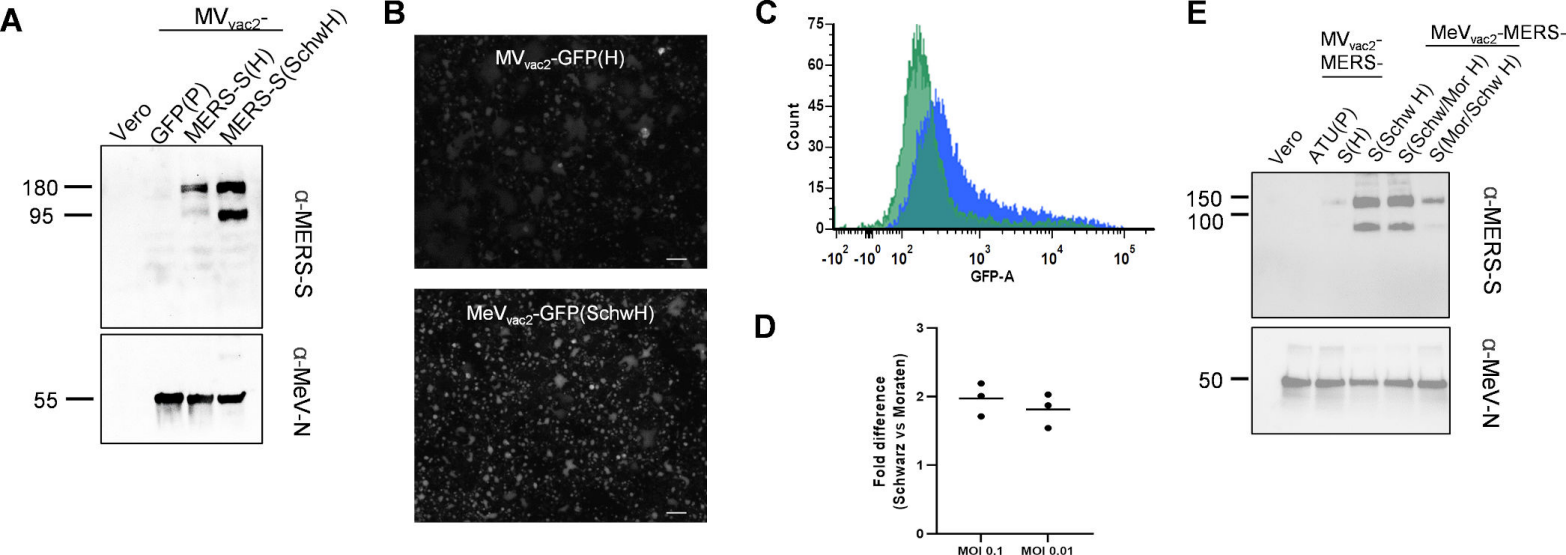
Effect of altered ATU sequences. (**A**) Immunoblot analysis of Vero cells 48 h after infection (MOI 0.03) with MeV_vac2_-GFP(P), MV_vac2_-MERS-S(H), or MeV_vac2_-MERS-S(SchwH) as depicted above lanes. Uninfected cells (Vero) served as a mock. Blots were probed using anti-MERS Spike antibody (upper blot) or mAb reactive against MeV-N (lower blot). (**B**) GFP fluorescence in Vero cells 48 h after infection (MOI 0.1) with either MV_vac2_-GFP(H) or MeV_vac2_-GFP(SchwH). (**C and D**) Quantification of GFP expression in Vero cells by FACS analysis. (**C**) Representative overlay of histograms of Vero cells infected with MV_vac2_-GFP(H) (green) or MeV_vac2_-GFP(SchwH) (blue). (**D**) Relative mean fluorescence intensities of Vero cells infected with MeV_vac2_-GFP(SchwH) (Schwarz) vs. MV_vac2_-GFP(H) (Moraten) 48 h after infection with two different MOIs as indicated. Single dots represent results of three independent experiments. (**E**) Immunoblot analysis of Vero cells 48 h after infection (MOI 0.03) with MeV_vac2_-ATU(P), MV_vac2_-MERS-S(H), MeV_vac2_-MERS-S(SchwH), MeVvac2-MERS-S(Schw/Mor H), or MeV_vac2_-MERS-S(Mor/Schw H) as depicted above lanes. Uninfected cells (Vero) served as mock. Blots were probed as described for panel A.

Immunoblot analysis indeed revealed higher expression of S in Vero cells infected by MeV_vac2_-MERS-S(SchwH) compared to MV_vac2_-MERS-S(H), both viruses just differing in the ATU region and analyzed 48 h after infection (MOI = 0.03) (Fig. 3A). This enhanced antigen expression neither impaired viral replication nor CPE in infected Vero cells (Fig. S3). For easier comparability and quantification of protein expression, we additionally used the eGFP reporter gene in the chimeric virus termed MeV_vac2_-GFP(SchwH) (Fig. S2) and analyzed infected Vero cell cultures side-by-side with control virus MV_vac2_-GFP(H) by fluorescence microscopy (Fig. 3B) and FACS analysis (Fig. 3C). As already evident from fluorescent microphotographs recorded with similar exposure times, the culture infected by the chimeric virus was fluorescing considerably brighter (Fig. 3B). This difference became quantified by flow cytometry for GFP expression (Fig. 3C). Calculating the fold-difference of the mean fluorescence intensity (MFI) of the histograms, the cultures infected with the chimeric MeV_vac2_-SchwGFP(H) expressed about twice as much GFP as cultures infected with MV_vac2_-GFP(H) during both high and low MOI infections (Fig. 3D).

To further narrow down which part of the Schwarz ATU site is responsible for enhanced antigen expression, we generated hybrid viruses containing either a genomic 5′ UTR of the Schwarz ATU site and a genomic 3′ UTR of the Moraten ATU site or vice versa to yield MeV_vac2_-MERS-S(Mor/Schw H) or MeV_vac2_-MERS-S(Schw/Mor H), respectively (Fig. S1 to S3). To assess the quantity of expressed S-antigen in direct comparison to MV_vac2_-MERS-S(H) with the Moraten ATU and MeV_vac2_-MERS-S(Schw H) with the complete Schwarz-ATU, lysates of infected Vero cells (MOI = 0.1) were checked by Western blot analysis for expression of MERS-CoV S and MeV N proteins (Fig. 3E). Thereby, considerably higher S expression was detected also for MeV_vac2_-MERS-S(Schw/Mor H) in addition to MeV_vac2_-MERS-S(SchwH), while S expression in cells infected with MeV_vac2_-MERS-S(Mor/Schw H) was just comparable to MV_vac2_-MERS-S(H) infection (Fig. 3E). Thereby, the determinants for higher expression of the antigen were traced back to the genomic 3′ UTR of the recombinant ATU as cloned in the Schwarz-strain-derived vaccine.

To additionally rule out an abundance of defective interfering RNA (DI RNA) as a potential cause for modulated viral RNA metabolism and antigen expression due to interference with vRNA metabolism, in general, we analyzed all MeV preparations for the abundance of DI RNA by RT-PCR (40). As a positive control for the abundance of DI-RNA, we employed a C-protein-deficient recombinant MeV, MeV-vac2-C^KO^(GFP) (40, 47). No analyzed vaccine preparations revealed the abundance of characteristic DI RNA (Fig. S4). Thereby, induction of DI RNA can be ruled out as causative for the observed differences in antigen expression. Therefore, the differences in expression of S could be attributed to the different ATU sequences of the used recombinant MeV constructs.

### Induction of antibody responses by vaccine candidates

**Fig 4:**
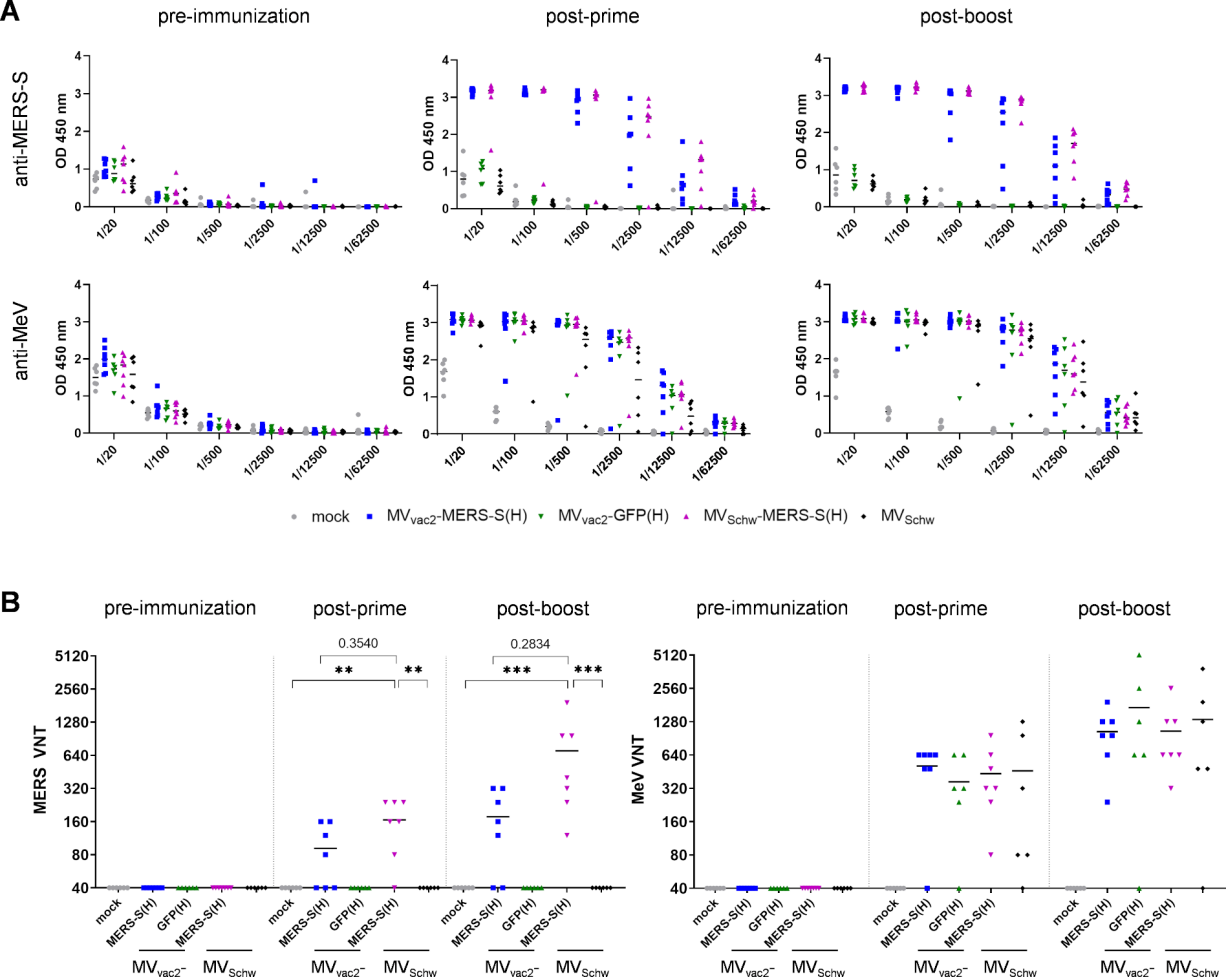
Induction of α-MERS-CoV S and α-MeV-specific antibodies. Sera of mice vaccinated on days 0 and 28 with indicated viruses were sampled on day 0 (pre-bleed), day 28 after prime immunization (post-prime), and day 49 after boost immunization (post-boost) and analyzed for antibodies specific for MERS-CoV S or MeV. Medium-inoculated mice served as mock. (**A**) Pan-IgG binding to recombinant MERS-CoV S (upper panel) or MeV bulk antigens (lower panel) was determined by ELISA via the specific OD 450 nm value. Depicted are individual animals of each cohort (*n* = 6–7), horizontal bars represent means. (**B**) VNTs in vaccinated mice for MERS-CoV (left panel) or MeV (right panel) were calculated as the reciprocal of the highest dilution abolishing infectivity. Dots represent single animals; the horizontal line represents the mean per group. The Y-axis starts at the detection limit; all mice at the detection limit had no detectable VNT. For statistical analysis of VNT data, Kruskal–Wallis test was performed in combination with Dunn’s multiple comparisons test to compare all pair means. **, *P* < 0.01; ***, *P* < 0.001. Gray circles, mock; blue squares, MV_vac2_-MERS-S(H); green triangles, MV_vac2_-GFP(H); magenta triangles, MV_SCHW_-MERS-S(H); black diamonds, MV_SCHW_.

After the demonstration of antigen expression for both recombinant vaccine candidates, we analyzed the impact on the induction of immune responses in the standard mouse model for MeV-derived vaccines, IFNAR^−/−^-CD46Ge mice. Mice were vaccinated in a prime-boost protocol with immunizations separated by 4 weeks and sacrificed 3 weeks after the booster immunization. Blood was drawn before prime and booster vaccinations and at the end of the experiment, and antibodies in sera were tested for binding antibodies (bAbs) (Fig. 4A) and neutralizing antibodies (nAbs) (Fig. 4B) targeting MERS-CoV or MeV. While all mice were seronegative before the first immunization, most animals, besides the mock control group, were reactive against MeV bulk antigens already after the first immunization. MeV nAb titers were in a similar range for all MeV-treated animals around a virus-neutralizing titer (VNT) of 400. Only one animal each in the vector control cohorts vaccinated with MV_vac2_-GFP(H) or MV_Schw_ did not react upon the first vaccination. bAb titers were slightly boosted by the second immunization, whereas nAb titers were considerably boosted with individual animals reaching a VNT in excess of 2,000. No evident difference was observed between the MeV vaccine cohorts in inducing anti-measles immunity in the mice.

For anti-MERS-CoV immunity, the picture was slightly different. Only the cohorts treated with MERS-vaccine candidates revealed induction of antibodies binding to S, with higher bAb titers induced in animals immunized with Schwarz-strain-derived MV_Schw_-MERS-S(H) especially after one immunization. The second immunization leveled the difference in bAb titers to some extent, with the MV_vac2_-MERS-S(H)-immunized animals profiting more from the boost. However, slight differences in nAb titers already evident after the first immunization became more pronounced. All animals in the MV_Schw_-MERS-S(H) group revealed a VNT above 120, with one animal reaching a VNT of nearly 2,000 (median VNT = 400). In contrast, two animals in the MV_vac2_-MERS-S(H) cohort remained below the LOD of VNT = 40, with just two animals revealing a VNT of 320 (median VNT = 160). Therefore, higher S antigen expression by the MV_Schw_-MERS-S(H) vaccine candidate correlated with more effective induction of antibodies targeting MERS-CoV S in the mouse model.

### Induction of T cell responses by vaccine candidates

**Fig 5:**
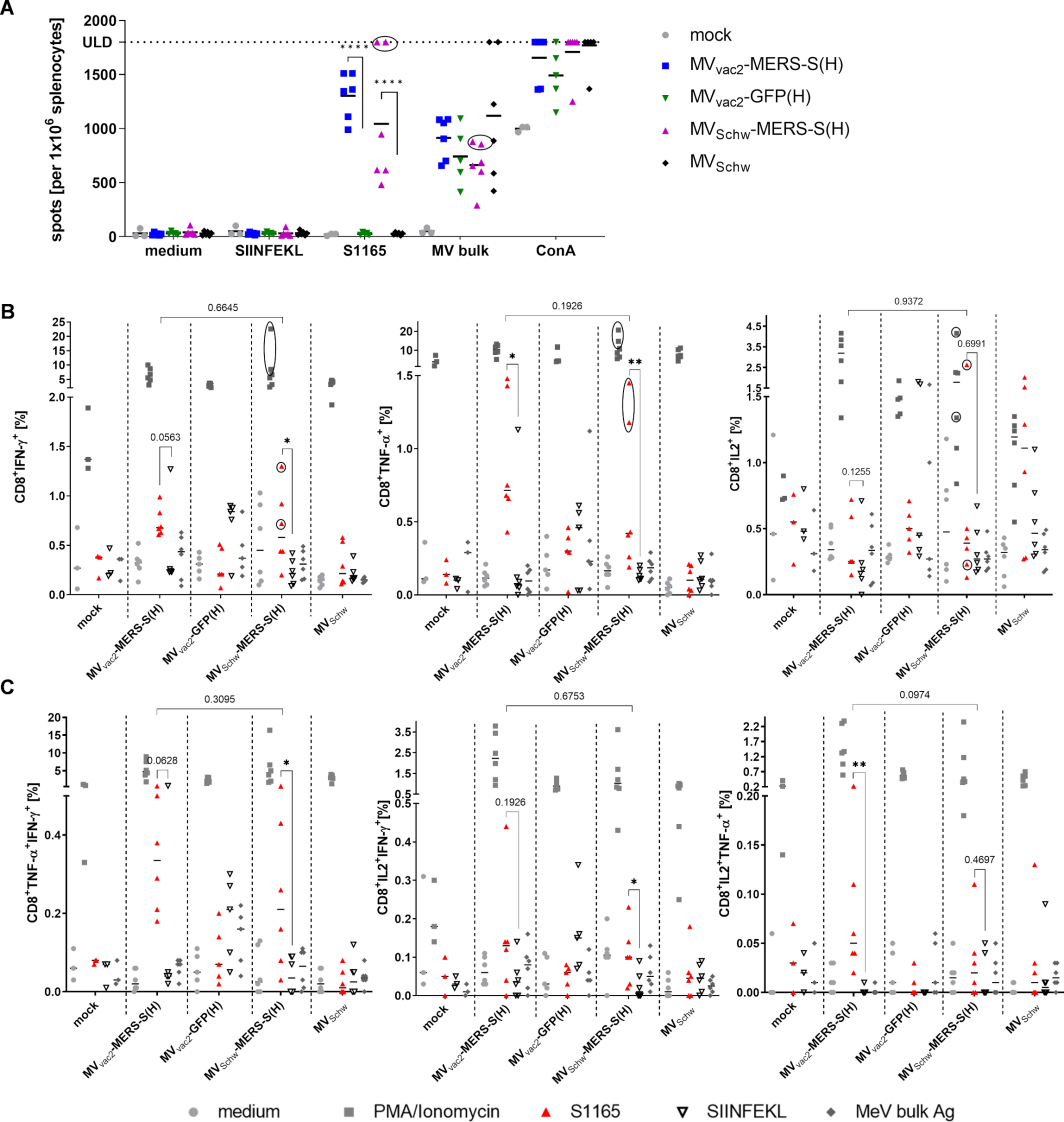
Reactivity of splenocytes after antigen-specific re-stimulation. (**A**) IFN-γ-ELISpot analysis using splenocytes of mice vaccinated on days 0 and 28 with the indicated vaccines, isolated 7 days after boost immunization, and after stimulation with the immunodominant MERS-CoV S peptide S1165 or the irrelevant control peptide SIINFEKL. To analyze cellular responses directed against MeV, splenocytes were stimulated with 10 μg/mL MeV bulk antigens or were left unstimulated as controls (medium). The reactivity of splenocytes was confirmed by Concanavalin A (ConA) treatment (10 μg/mL). The number of cells per 1 × 10^6^ splenocytes represents the number of cells expressing IFN-γ upon re-stimulation. Dots represent individual animals, horizontal bars mean per group (*n* = 5–6). Spot counts above the countability of the software were set to the upper detection limit (ULD). For statistical analysis of grouped ELISpot data, two-way ANOVA analysis was applied with paired Tukey’s multiple comparison test as post hoc test. ****, *P* < 0.0001. Encircled animals did not react to the first immunization (see Fig. S3B). (**B and C**) Harvested splenocytes of vaccinated mice (same as depicted in panel A) were re-stimulated and subjected to intracellular staining (ICS) for IFN-γ, TNF-α, and IL-2, and stained for extracellular T-cell markers CD3 and CD8 for flow cytometry analysis. Quantification of flow cytometry data of (**B**) single cytokine and (**C**) double cytokine CD8^+^-positive T cells after incubation with the indicated stimuli; reactivity of splenocytes was confirmed by Tetradecanoylphorbol-acetate and Ionomycin (TPA/Iono) treatment (10 μg/mL). Dots represent individual animals, horizontal bars represent the median. For statistical analysis, the Mann–Whitney test was performed to compare two stimuli within the same vaccine cohort or the same stimuli between two vaccine groups: *, *P* < 0.05; **, *P* < 0.01. The animals marked with the circle did not react to the prime immunization (see panel A).

Besides antibodies, T-cell responses can play a crucial role in protecting against disease. Therefore, we determined cellular immunity against MERS-CoV and as a control against MeV by our MERS vaccine candidates. For this purpose, IFNAR^−/−^-CD46Ge mice were vaccinated as described above, but immunized animals were sacrificed 7 days after the second immunization for the determination of antigen-specific T-cell reactivity. Freshly isolated splenocytes were subjected to IFN-γ ELISpot analysis (Fig. 5A) or stained for cytokine expression via intracellular cytokine staining (ICS) and surface markers and analyzed by flow cytometry analysis (Fig. 5B and C) for expression of IFN-γ, TNF-α, and IL-2 in CD8^+^ or CD4^+^ splenocytes. As a control for vaccination, bAb titers in sera of these mice after prime and final bleed were determined (Fig. S5).

IFN-γ ELISpot revealed a comparable general reactivity of splenocytes in all vaccine-treated cohorts after stimulation with concanavalin A. Only the naïve mock cohort revealed a trend for lower spot counts. Also, after stimulation with MeV bulk antigens, no considerable differences between the four vaccine-treated cohorts became evident, with mean spot counts between 300 and 1,800 reactive cells per 10^6^ splenocytes. In contrast, only the animals immunized with MERS-vaccine candidates demonstrated specifically reactive T cells after stimulation with the immunodominant S1165 peptide, the stimulatory capacity of which was controlled by the in this context irrelevant SIINFEKL peptide. Here, the observations for antibody responses were reversed, with MV_vac2_-MERS-S(H)-immunized animals revealing a trend for induction of higher numbers of S-reactive T cells. Remarkably, two individual animals in the MV_Schw_-MERS-S(H)-treated group reversed this trend with spot counts exceeding the upper limit of detection (ULD). Closer examination of these two animals revealed that they were exactly those that also stood out in the antibody response analysis after the first immunization (Fig. S5): these are the two animals in the MV_Schw_-MERS-S(H) cohort that showed no or very low bAb titers against MeV and MERS-CoV S after the prime, indicating a vaccination failure during the prime immunization. Therefore, our data provide anecdotal evidence for more effective induction of cargo antigen-specific T-cell responses by the MeV-derived MERS vaccine candidates after a single-shot vaccination.

Interestingly, these reactivities were not too short-lived. When sacrificing the animals for determining humoral responses 3 weeks after the second vaccination, we also prepared the splenocytes of these animals and subjected those to ELISpot analysis (Fig. S6). Even though 3 weeks post-vaccination considerably exceeds the optimal time for antigen-specific T-cell recall after immunization, the observed responses were similar to those observed 7 days after the boost. This data set provides evidence for robust and reproducible induction of stable T-cell reactivities in immunized animals by both MeV-derived MERS vaccine candidates.

ICS analysis confirmed the ELISpot data for IFN-γ (Fig. 5B, left), but also provided evidence for induction of TNF-α (Fig. 5B, middle) or IL-2 (Fig. 5B, right) by MERS-CoV S-specific re-stimulation in CD8^+^ splenocytes of animals that had been vaccinated with the MeV-derived MERS vaccine candidates. Remarkably, this analysis also provided evidence for multi-reactive T cells expressing more than one cytokine after re-stimulation (Fig. 5C) for both MERS vaccine candidates. Again, a slight trend for higher numbers of S-specific T cells was observed in the MV_vac2_-MERS-S(H) immunized cohort. Re-stimulation of CD4^+^ splenocytes was generally less efficient, but the highest reactivity was found for TNF-α secretion after stimulation of splenocytes with MeV bulk antigen in MeV-immunized animals (Fig. S7).

Taken together, both MERS-vaccine candidates showed efficient induction of humoral and cellular immune responses against MERS-CoV S, with MV_Schw_-MERS-S(H) inducing higher antibody titers and MV_vac2_-MERS-S(H) showing a trend for better T-cell responses. In the absence of correlates of protection against MERS-CoV, we therefore needed to test the protective efficacy of both vaccines side-by-side.

### Setup of MERS-CoV challenge in IFNAR^−/−^-hDPP4^+/−^ mice to be vaccinated with recombinant MeV

For susceptibility to MERS-CoV, mice must express the primary entry receptor of MERS-CoV, human DPP4 (also known as CD26), as a transgene. hCD26 tg mice are very susceptible to MERS-CoV and develop a lethal infection upon intranasal (i.n.) inoculation (45). However, fully immune-competent mice as the background of those do not confer considerable MeV replication. Usually, double-modified IFNAR^−/−^-CD46Ge mice are used for testing recombinant MeV in mice (11, 44). More recently, it was shown that the decisive factor for MeV-permissiveness of those mice is not the hCD46 receptor transgene, but rather the knockout of the type-I interferon receptor (48). We reasoned that crossing the hDPP4 transgene into IFNAR^−/−^ mice would yield a mouse line, IFNAR^−/−^-hDPP4^+/−^, which can be vaccinated by recombinant MeV, is susceptible, and could therefore be used for challenge experiments with MERS-CoV.

**Fig 6:**
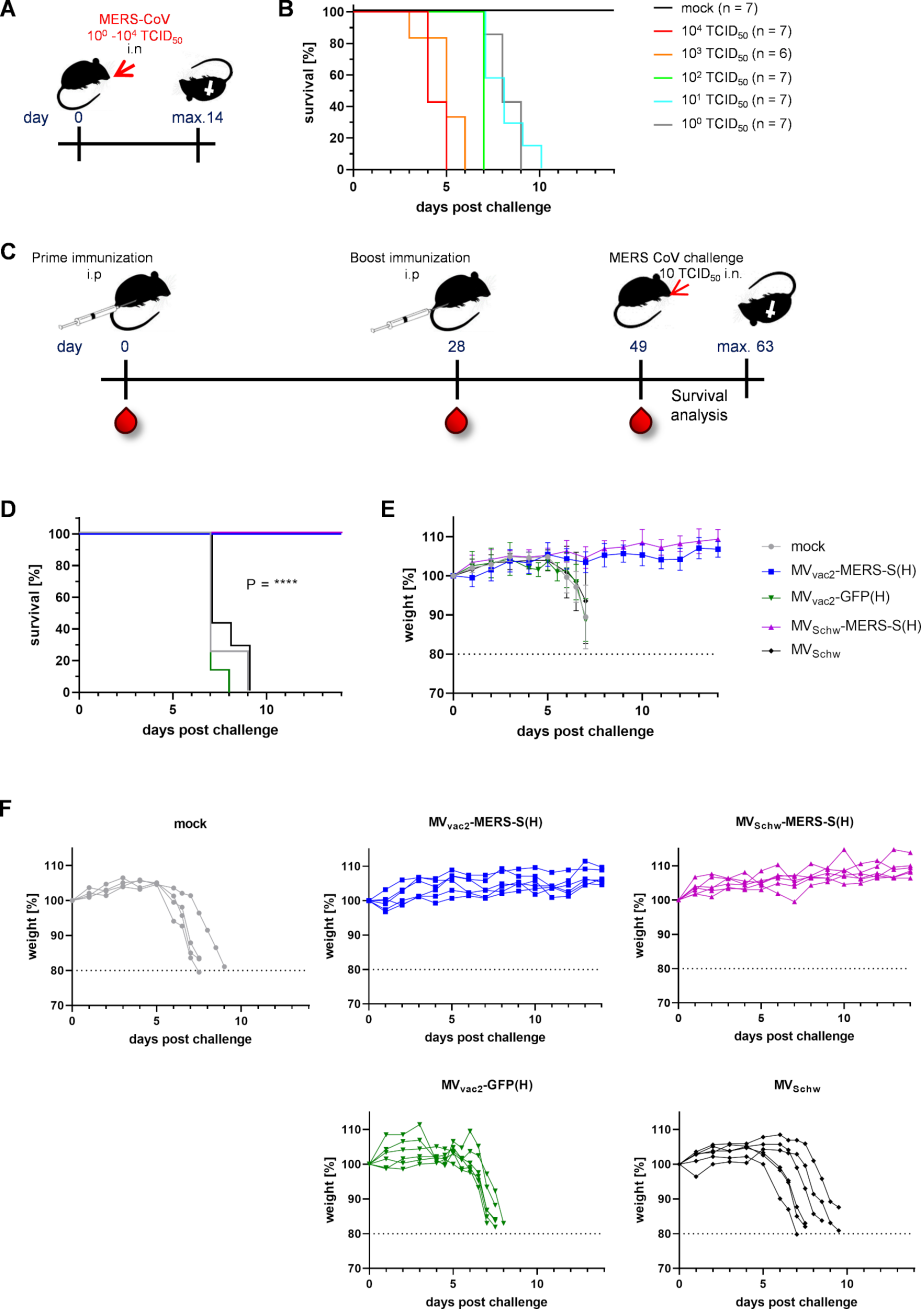
MERS-CoV challenge experiments. (**A**) Challenge scheme used for *in vivo* titration of challenge dose of intranasal MERS-CoV used in IFNAR^−/−^-hDPP4^+/−^ mice ranging between 10^0^ and 10^4^ TCID_50_/mouse. (**B**) Results of dose titration. Kaplan–Meier survival curve for indicated dose cohorts (*n* = 6–7). For survival, the weight cutoff of ≥ 80% of initial body weight was used. (**C**) Vaccination scheme used in challenge experiments shown in (**D–F**). MERS-CoV challenge of vaccinated mice employing a dose of 10^1^ TCID_50_/mouse. (**D**) Survival and (**E**) weight loss in cohorts of mice vaccinated as indicated (*n* = 4, mock; *n* = 6, all other). (**F**) Weight of individual animals shown in (**D and E**) over time after infection. Gray circles, mock; blue squares, MV_vac2_-MERS-S(H); green triangles, MV_vac2_-GFP(H); magenta triangles, MV_Schw_-MERS-S(H); black diamonds, MV_Schw_. ****, *P* < 0.0001 (log-rank [Mantel–Cox]).

Before breeding this line, we wanted to ensure that IFNAR^−/−^ mice are indeed a suitable model for vaccination with MeV-derived MERS vaccines. For this purpose, we vaccinated in parallel parental immune-competent C57/BL6 mice, IFNAR^−/−^ mice, and IFNAR^−/−^-CD46Ge mice with recombinant measles vaccine MV_vac2_-ATU(P) and MV_vac2_-MERS-S(H) in a prime-boost schedule as depicted in Fig. 6C. Also, the splenocytes were assessed on final day 49, again, for induction of measles- and MERS-CoV S-specific immune responses. Our data indeed confirm the study by Mura et al. (48): while both humoral and cellular immune responses against S were virtually indistinguishable between IFNAR^−/−^ and IFNAR^−/−^-CD46Ge mice, induction of nAbs and S-specific T cells was significantly lower in parental C57/BL6 mice (Fig. S8). Thus, we concluded that IFNAR^−/−^-hDPP4^+/−^ mice should be a suitable model for our purpose.

After the IFNAR^−/−^-hDPP4^+/−^ mice were available, we titrated the i.n. challenge dose in 10-fold increments between 10^4^ and 10^0^ (i.e., 1) TCID_50_ to identify the LD_50_ of MERS-CoV in these mice (Fig. S9). While mock-treated animals gained weight over the course of the experiments, all mice in infected cohorts started at one point to dramatically lose weight (Fig. S9B) and had to be sacrificed over the next 3 days (Fig. S9B). This effect was dose-dependent, with lower dose-infected mice becoming ill at later time points (Fig. S9A). Intriguingly, all mice infected with only one TCID_50_ had to be sacrificed eventually, demonstrating that the LD_50_ of MERS-CoV in IFNAR^−/−^-hDPP4^+/−^ mice is at even lower dose levels. To eventually re-titrate the challenge virus and to have a robust challenge dose, we decided to set the challenge dose in vaccinated mice to 10 TCID_50_ MERS-CoV.

### Protection of IFNAR^−/−^-hDPP4^+/−^ mice against lethal challenge

To utilize the newly established MERS-CoV challenge model, IFNAR^−/−^-hDPP4^+/−^ mice were vaccinated in a prime-boost setup with an interval of 4 weeks, and challenged 3 weeks after the boost vaccination (Fig. 6C). Mice were bled before the first vaccination, the second vaccination, and challenge to control for efficient immunization. Serology revealed that all mice had been properly immunized, with the previously observed higher antibody responses against MERS-CoV S in the cohort vaccinated with the Schwarz-strain-derived MV_Schw_-MERS-S(H) (Fig. S10).

Upon challenge, mock-immunized mice started to develop symptoms 5 days after infection and had to be killed by day 9 (Fig. 6D and F, left), as observed for naïve animals in the dose-titration study (Fig. 6B). The animals in the control cohorts that were vaccinated with MeV strain-specific control viruses (MV_vac2_-GFP(H) for Moraten and MV_Schw_ for Schwarz) showed the same patterns, demonstrating no unspecific effects of measles vaccination on the infection with MERS-CoV. However, all mice in the vaccine cohorts were completely protected (Fig. 6D and F) and gained weight over the experiment. Just one animal in the MV_Schw_-MERS-S(H) cohort dropped below its initial weight around day 7 post-infection, but did not show any other symptoms and regained its body weight fully to match all other MERS-vaccinated mice, thereafter. Thus, our data provide evidence for the complete protection of vaccinated animals by both Moraten- or Schwarz-strain-derived MERS vaccine candidates in an otherwise uniformly lethal, robust challenge model.

## DISCUSSION

We demonstrate in this study that minor differences in the backbone of an otherwise highly similar MeV vector can influence the expression of additionally encoded foreign antigens. These differences could be confirmed to be due to alterations of genomic 3′ UTR signals in recombinantly inserted additional transcription units that allow the expression of these extra genes. Moreover, these differences in antigen expression seem to modulate the induced immune responses, favoring either humoral or cellular responses to the very same antigen. In agreement with data presented before (48), we then provided further evidence for the suitability of IFNAR^−/−^ mice as host species to analyze MeV-derived vaccines and established IFNAR^−/−^-hDPP4^+/−^ mice as a model for fulminant MERS-CoV-induced disease. In this model, both candidates robustly protected transgenic mice against an otherwise uniformly lethal challenge with highly pathogenic MERS-CoV despite their slightly different immunogenicity.

Those slight differences in immunogenicity are most likely directly connected to the different amounts of transgene expressed by vaccine-infected cells. For the MeV vector system, expression of the HBsAg from three different genomic positions (post-P, post-H, or post-L) with a corresponding gradient of antigen expression has been shown for Moraten-strain-derived vaccines (20). Use of these three different recombinant vaccines to immunize IFNAR^ko^-CD46Ge mice revealed differential induction of antibodies binding to HBsAg, correlating with the amount of expressed antigen. As a control, a vectored MeV derived from another attenuated strain, Edmonston-Tag, encoding the HBsAg also from an ATU in the post-P position showed comparable titers as the Moraten strain with HBsAg in post-P, MVvac2-(HBsAg)P. Upon translating these findings from mice to rhesus macaques, only two out of four animals vaccinated with MVvac2-(HBsAg)*P* seroconverted against HBsAg, but no animals vaccinated with MVvac2-(HBsAg)H (with lower antigen expression from the post-H position). These data are consistent with our observations of modulated humoral responses due to the amount of antigen expression, especially as the differences in antigen expression levels were determined to be in the range of a factor of 2 in the former study, as well (20). However, further increase in relative HBsAg expression using an over-attenuated MeV encoding HBsAg in the first position of the vaccine genome, MVvac2-(HBsAg)N, did not result in higher anti-HBsAg bAb titers in mice, nor in better seroconversion rates of macaques (49). Thus, the correlation of immunogenicity to the amount of antigen may become saturated at one point, or the antigen effect may have become counteracted by the slower vaccine replication in the latter study. Unfortunately, no results for T-cell responses against HBsAg were presented.

Expression of such additional antigens in recombinant MeV-infected cells becomes feasible by encoding additional transcription units in the MeV genome (5). As already outlined in the introduction, the conserved transcriptional STOP-START sequences for the viral polymerase are duplicated between two viral transcription cassettes and allow insertion of an additional ORF, in between, the mRNA of which becomes transcribed in infected cells. However, due to the different cloning history and introduction of different restriction enzyme recognition sites, which facilitate cloning of ORF-sequences, there are slight differences between the MeV backbones used for generating recombinant MeV. As shown in Fig. 1A and Fig. S2, there is a difference of 28 nts in the 3′ and 5′ UTRs of Moraten-strain-derived MV_vac2_-GFP(H) (27, 50) and Schwarz-strain-derived MV_Schw_-ATU3-GFP (19), while the rest of the backbone is highly conserved. For AIK-C-strain-derived vaccines, a cloning strategy was chosen that inserted the antigen after the natural STOP-START sequence, followed by the duplicated START-STOP sequence, thereby generating the same overall pattern, but different sequences, in detail (21, 51).

Nevertheless, for Moraten- and Schwarz-strain-derived candidates analyzed in this study, the transcription rates of the additional mRNAs in the respective ATUs seem to be similar, as evident by a similar relative abundance of S mRNA early after infection (Fig. 2). Unimpaired vaccine-like replication of both studied recombinant vaccines (Fig. 1B) furthermore implies a rather conserved transcription and transcriptional gradient of all virus ORFs for those vaccine candidates. Therefore, the core ATU elements utilized in MV_vac2_-MERS-S(H) are obviously sufficient to comply with gene transcription by the viral polymerase complex. This would be in accordance with constant relative transcription rates observed for unmodified MeV (52). Moreover, the differential abundance of S mRNA in infected cells over time suggests a relative accumulation of S mRNA for MV_Schw_-MERS-S(H). In the view of comparable gene transcription, such accumulation could be explained by different half-lives of mRNA species under analysis. Viral mRNA is indeed degraded in MeV-infected cells (52). Since all other factors in the cells infected by MV_vac2_-MERS-S(H) or MV_Schw_-MERS-S(H) are similar, the UTRs of the S mRNA expressed from MV_Schw_-MERS-S(H) are the most likely explanation for the differential accumulation of S mRNA, as observed. Indeed, a Moraten-like virus encoding MERS-CoV S in an ATU as used in the Schwarz backbone revealed higher expression of S than the parental Moraten-derived MV_vac2_-MERS-S(H), which is also true if just the genomic 3′ UTR was similar to the recombinant Schwarz strain MeV. Moreover, generation of a marker protein-encoding virus, MV_vac2_-GFP(SchwH), which encodes the marker protein GFP in the same configuration, enabled quantification and further demonstrated the causal relationship between transgene expression and ATU/UTR sequences, more precisely the 5′ UTR of the transcribed mRNA (Fig. 3).

The potential of mRNA’s 3′ UTRs (53, 54) to enhance protein expression has been demonstrated using *in vitro*-transcribed RNA. Also, mRNA’s 5′ UTR sequences can affect gene expression, but rather by modulating translational efficacy (55). For MeV and other closely related morbilliviruses, an unusually long stretch of UTRs is located between the ORFs encoding the matrix protein M and the fusion protein F (56–58). These regions regulate the expression of M and F in addition to the transcriptional gradient of morbilliviruses. Substitution or deletion of these regions modulates the amount of the corresponding viral mRNAs as well as of viral protein (59), demonstrating considerable impact of UTRs also on MeV gene expression. Therefore, an enhanced stabilizing effect of UTR sequences, especially the genomic 3′ UTR found in the recombinant Schwarz vaccine, can be postulated. Interestingly, exactly the opposite was shown for the mRNA 5′ UTR of the F mRNA, which seems to destabilize the RNA compared to a UTR-deleted version of the gene when transiently expressed, while the corresponding M gene expression remains unaltered (53). Therefore, our data provide the first evidence for the general impact of mRNA 5′ UTRs of viral mRNA using different, foreign ORFs in the context of replicating MeV. However, the 5′-cap of mRNAs (60) as well as the length of their poly-A tail (53) modulates the stability of mRNA and therefore the translational efficacy. During paramyxoviral gene expression, both processes are co-transcriptionally performed by the viral polymerase complex (61). While the transcriptional activity of this vRNP complex seems unaltered (as discussed above), we cannot rule out that capping and polyadenylation activity could be modulated depending on the exact ATU sequences in the recombinant virus’ genomes. The exact mechanism of viral mRNA stabilization by the genomic 3′ UTR sequences of the viral transcription cassettes will be a matter of future studies.

Despite the differences observed for both MeV-derived vaccine candidates, mice immunized with either vector reveal robust and full protection in our setup when challenged with a dose higher than 10 LD_100_. This means that both vectors exceed the threshold of immune reactions required to protect, despite the bias of the Moraten-strain-derived MV_vac2_-MERS-S(H) to drive higher T-cell immunity, whereas Schwarz-strain-derived MV_Schw_-MERS-S(H) triggered more potent humoral responses. No definitive correlates of protection (CoP), which indicate the immune reactions that protect against disease, have been defined for MERS-CoV, so far (62). Also, no clear-cut CoP has been defined for the other two beta-CoVs that cause potentially lethal disease in human patients, that is, SARS-CoV and SARS-CoV-2. Only recently, partial protection of hamsters by high-titer neutralizing convalescent plasma (63) or a combination of memory CD8^+^ T-cell and B-cell responses correlated with protection in patients after vaccination with the COVID-19 mRNA vaccine (64). Moreover, all of our data have been generated in animal models, that is, transgenic mice. Those models can hint, but not accurately predict the situation in human beings. Clinical studies will be needed to demonstrate the translatability of our findings.

In any case, a vector system such as MeV that stimulates both arms of the adaptive immune system may be highly suitable to protect against such CoV-induced diseases. Indeed, also for SARS-CoV (65, 66), and especially SARS-CoV-2 (12–14, 17, 67), different animal models indicate the protective potential of MeV as a vector system against disease induced by highly pathogenic CoVs. Also, our analyses reveal full protection of vaccinated mice by both vaccine candidates, irrespective of the slightly different immune responses, and the high susceptibility of the newly established challenge model in IFNAR^−/−^-hDPP4^+/−^ mice showing fulminant disease. The increased susceptibility of IFNAR^−/−^-hDPP4^+/−^ mice for MERS-CoV, as compared to the parental L52 hCD26/DPP4 transgenic mice with an LD_50_ of 10 TCID_50_ (68), is not uncommon for viral infections in mice lacking the type-I IFN receptor. The protective role of a functional type I IFN system became evident in early studies after the availability of IFNAR knockout mice. These studies revealed a more than 10^6^-fold higher susceptibility for vesicular stomatitis virus (VSV) or Semliki Forest virus (SFV) than the parental wt mice (42). However, the picture is somewhat different for SARS-CoV during mouse infection. Using mouse-adapted SARS-CoV, pathogenesis in mice is independent of Type I IFN receptor (69), and dysregulated type I IFN reactivity can cause even higher pathology (70).

In summary, our data provide evidence that minor differences between the backbones of MeV vaccine vectors may modulate the amount of antigen, which is expressed in vector-infected cells. In our case, this modulation is most likely due to differential half-lives of mRNA species caused by the 5′ UTR of the resulting mRNA, indicating that the ORF-flanking regions can be used to fine-tune gene expression. While different amounts of foreign antigen correlate with a bias for stronger humoral or cellular adaptive immune responses, both Moraten- and Schwarz-strain-derived MERS vaccine candidates showed impressive protection in a challenge model mirroring fulminant disease. Therefore, both candidates seem equally well-suited and support the measles vaccine vector technology, inducing both arms of the immune system, in principle. This becomes especially relevant in the absence of correlates of protection, which is the *a priori* situation during newly emerging infections.

## Data Availability

The complete vRNA genome of MV_vac2_-MERS-S(H) was deposited to the NCBI database and is available under BioProject accession no. PRJNA1438010. All other data generated or analyzed during this study are included in this published article and its supplemental material.

## SUPPLEMENTAL MATERIAL

Supplemental material jvi.00644-26-s0001.pdf Tables S1 and S2; Fig. S1 to S10.

ASM does not own the copyrights to Supplemental Material that may be linked to, or accessed through, an article. The authors have granted ASM a non-exclusive, world-wide license to publish the Supplemental Material files. Please contact the corresponding author directly for reuse.
