# Supplementary material for: Minor differences in the untranslated regions of measles vector additional transcription units are reflected by differential immunogenicity of encoded MERS-CoV Spike antigen
Source: J Virol. 2026 Jul 1;100(7):e00644-26. doi: 10.1128/jvi.00644-26 (PMC13386834; doi:10.1128/jvi.00644-26)
Supplement: Supplemental material — Tables S1 and S2; Fig. S1 to S10. [file jvi.00644-26-s0001.pdf]

Supplemental Material

Tiwarekar, Ebenig et al.

Minor differences in the untranslated regions of measles vector additional transcription units are reflected by differential immunogenicity of encoded MERS-CoV Spike antigen

Tiwarekar et al. Suppl. Table S1 – Primer used for cloning chimeric ATUs.

Chimera	Forward primer	Reverse primer
Moraten/ Schwarz	5'- GCG CGC AAC GCG TCG TAC GTC GCG AAT GAT CCA CTC CGT GTT TCT GCT GA -3'	5'-atg aga cgt cta agc gct gcg cgc tca TCA GTG CAC ATG CAC TTT GTG AGG TTC -3'
Schwarz/ Moraten	5'-gcg cgc acg cgt ACG ATG ATC CAC TCC GTG TTT CTG CTG A-3'	5´-atg aGA CGT Cgc gcg cta cgt atc gcg aTC Atc aGT GCA CAT GCA CTT TGT GAG GTT C -3'

Tiwarekar et al. Suppl. Table S2 – Primer used for qRT-PCR.

Name	Sequence	Reference
MV_{vac2}-N forward	5'-AGT GAG AAT GAG CTA CCG- 3'	(71)
MV_{vac2}-N reverse	5'-TGT CTA GGG GTG TGC C-3'	(71)
MV_{vac2}-H forward	5'-ACA TAC CTA CCT GCG G-3'	(71)
MV_{vac2}-H reverse	5'-AGT AAG AAA ATG AGC GGC-3'	(71)
hGAPDH forward	5'-GCC TTC CGT GTC CCC ACT GC-3'	(72)
hGAPDH reverse	5'-CCT CCG ACG CCT GCT TCA CC-3'	(73)
MERS-S #5 forward	5'-GTT CCC ATA TCA AGG CGA CCA CG-3'	n.a.
MERS-S #5 reverse	5'-CGT TGG CGA ACT GCT TCA CGT C-3'	n.a.

Tiwarekar et al. Suppl. Figure S1 – NGS coverage of vaccine virus

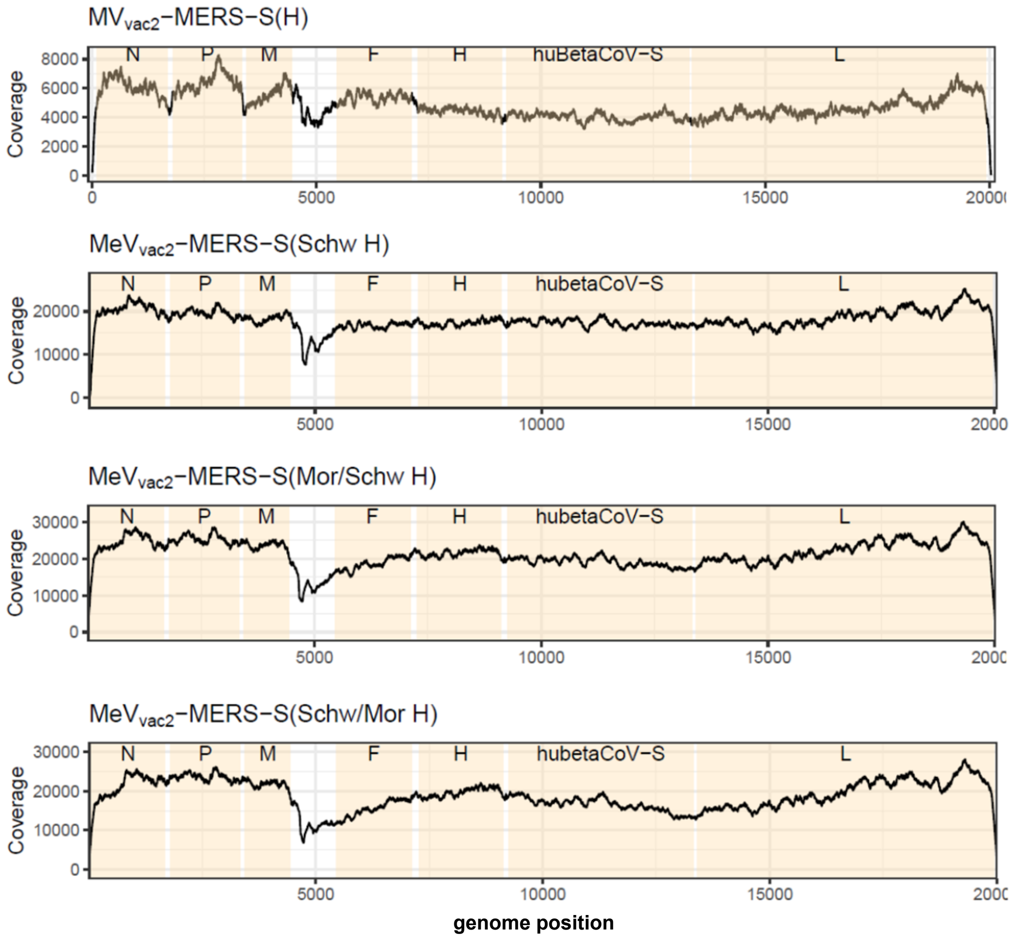

Suppl. Fig. S1: Coverage of different MeV.derived MERS-vaccine candidate genomes during next generation sequencing. Schematic depiction of read frequency at each position of the vaccine viruses´ genome. Beige areas indicate respective viral coding sequences, white areas indicate intergenic regions and untranscribed terminal regions (UTRs) of the genome. Coverage across the genome was sufficient for variant detection and reflects the transcription gradient typically observed in measles virus total RNAseq data. Since the majority of reads are mRNA-derived, low read numbers decrease between the coding regions and generally towards the 5’ end.

Tiwarekar et al. Suppl. Figure S2 – Alignment of ATU regions

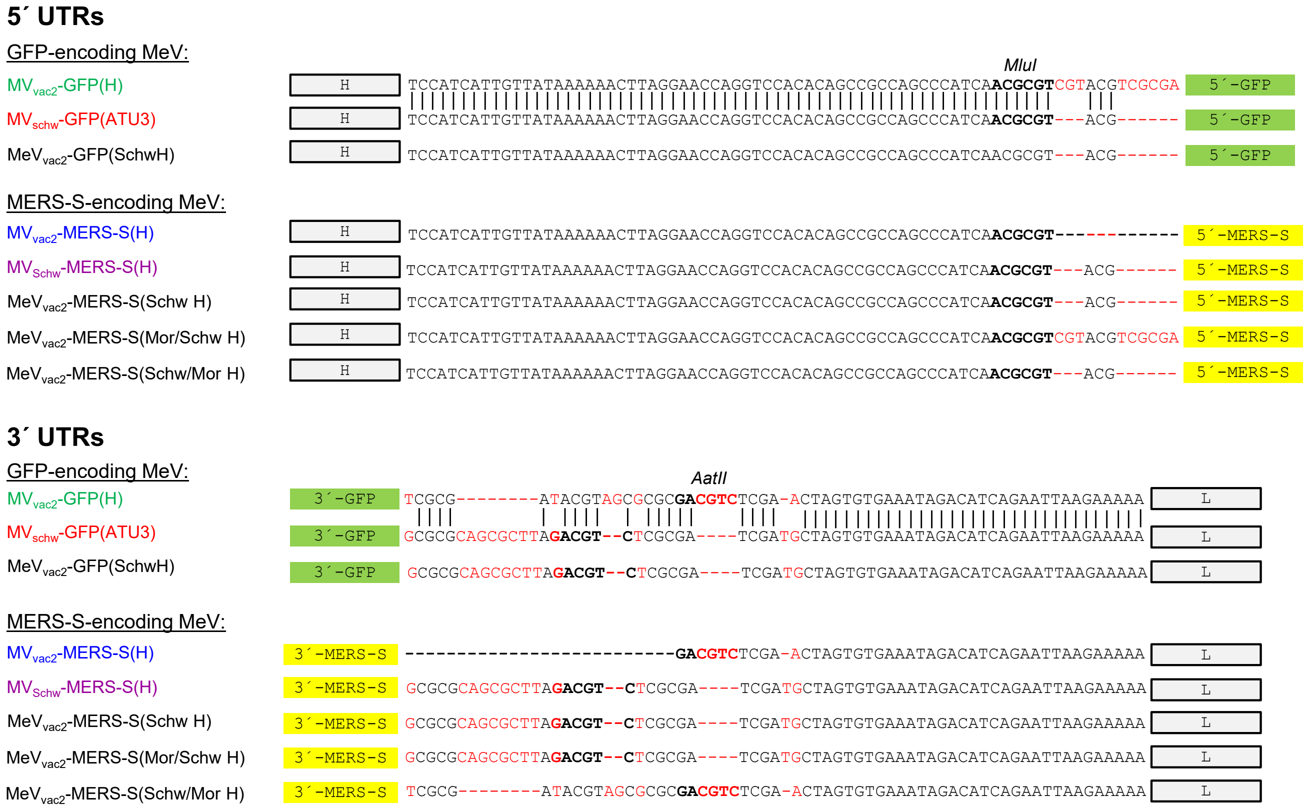

Suppl. Fig. S2: Head-to-head sequence comparison of the ATU sites of recombinant MeVs used in this study. ATU sequences of different recombinant MeV used in this study or available as references from database were aligned based on the genomic MeV sequence and respective differences are highlighted in red. Non-viral GFP and MERS-S ORFs are indicated sense direction of respective mRNA. Restriction sites used for cloning the Moraten strain-derived MeV are indicated above the sequence in italics. Flanking ORFs encoding MeV hemagglutinin (H) and polymerase (L) are indicated by grey boxes.

Tiwarekar et al. Suppl. Figure S3 – Characterization hybrid vaccines

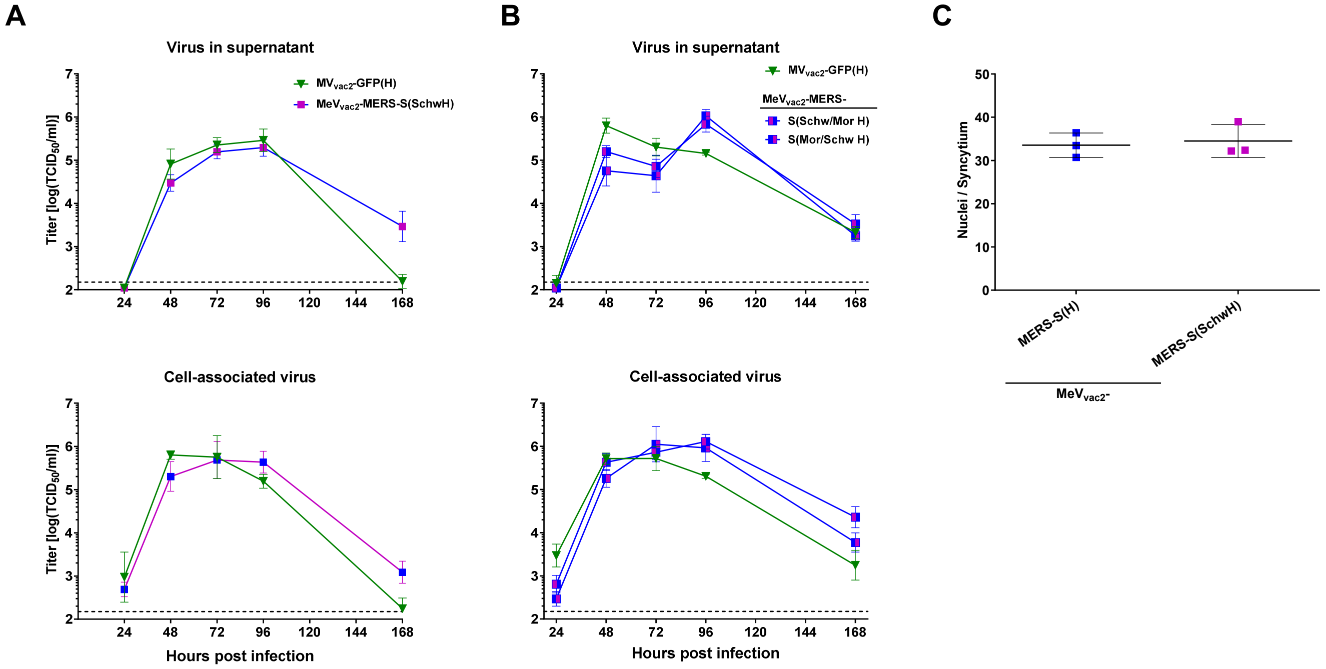

Suppl. Fig. S3: No changes in virus growth by enhanced antigen expression. Analysis of (**A, B**) replication kinetics and (**C**) CPE of Vero cells infected with the indicated vaccine viruses reveals no differences due to different amounts of S expressed in infected cells. Growth kinetics of recombinant MeV on Vero B4 cells infected at an MOI of 0.03 with (**A**) MeV$_{vac2}$-MERS-S(Schw H), (**B**) MeV$_{vac2}$-MERS-S(Schw/Mor H), MeV$_{vac2}$-MERS-S(Mor/Schw H) or control virus MV$_{vac2}$-GFP(H). Titers of samples prepared at indicated time points post infection were titrated on Vero B4 cells. (**C**) CPE induced in Vero B4 cells infected with the indicated vaccine viruses at an MOI of 0.01 for 30 h. Nuclei of 20 randomly selected syncytia were counted for each of 3 independent experiments. Means of each experiment are displayed, horizontal bar denotes overall mean, error bars SD.

Tiwarekar et al. Suppl. Figure S4 – DI RNA analysis

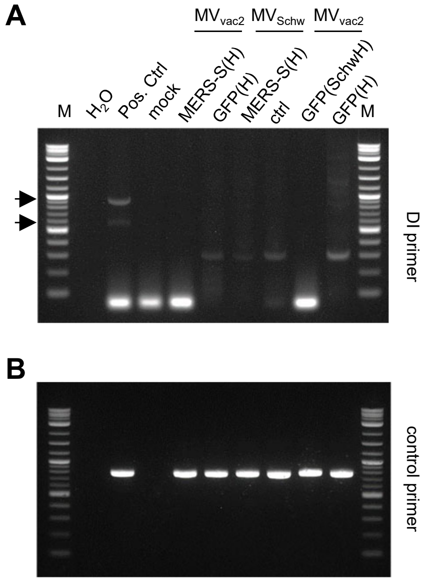

Suppl. Fig. S4: Absence of DI RNA in MeV-derived vaccine virus particle preparations. Analysis of differential abundance of defective interfering RNA in preparations of MeV-derived vaccines (**A**) RT-PCR with primers detecting defective interfering copy-back RNA in MeV vaccine preparation. Arrows indicating expected bands for DI RNA in positive control (MeV-vac2-C^{KO}(GFP)) particles (**B**) Control RT-PCR using primers binding in MeV RNA region to demonstrate intact viral RNA as template for RT-PCR reactions. H_2O, water control for RT-PCR with no template; Pos. Ctrl., MeV-vac2-C^{KO}(GFP); mock, RNA of uninfected Vero cells; M, marker.

Tiwarekar et al. Suppl. Figure S5 – Serology of mice used for analysis of cellular immune responses

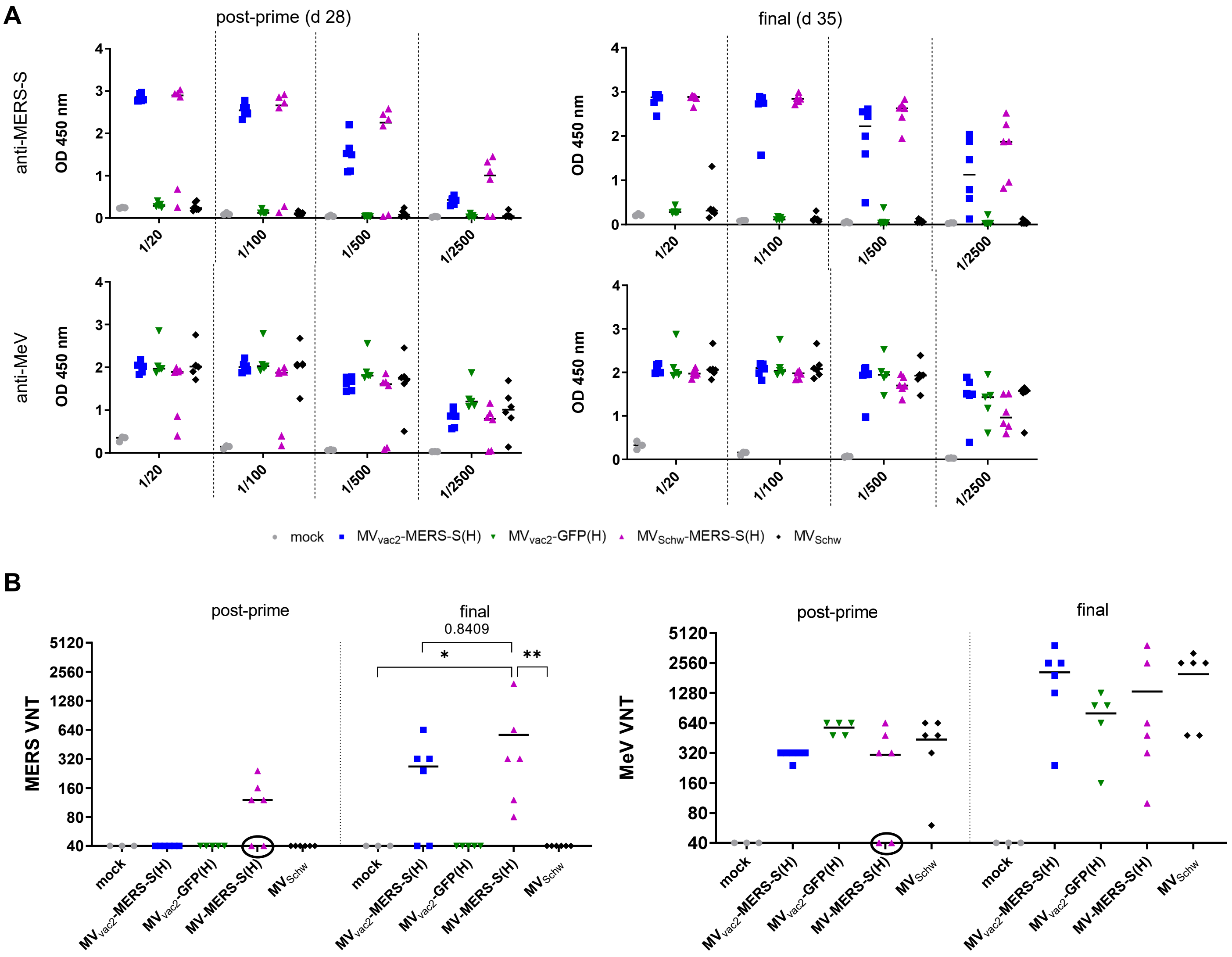

Suppl. Fig. S5: Induction of α-MERS-CoV S and α-MeV specific antibodies in mice used for analysis of cellular immune responses. Sera of mice (also depicted in Fig. 4) vaccinated on days 0 and 28 with indicated viruses were sampled on day 28 after prime- (post-prime) and day 35 after boost-immunization (final) and analyzed for antibodies specific for MERS-CoV S or MeV. Medium-inoculated mice served as mock. (**A**) Pan-IgG binding to recombinant MERS-CoV S (upper panel) or MeV bulk antigens (lower panel) were determined by ELISA via the specific OD 450 nm value. Depicted are individual animals of each cohort (n = 3 - 6), horizontal bars represent means. (**B**) Virus neutralizing titers (VNT) in vaccinated mice for MERS-CoV (left panel) or MeV (right panel) were calculated as reciprocal of the highest dilution abolishing infectivity. Dots represent single animals; horizontal line represents the mean per group. Y-axis starts at detection limit; all mice at detection limit had no detectable VNT. Grey circles, mock; blue squares, MV_{vac2}-MERS-S(H); green triangles, MV_{vac2}-GFP(H); magenta triangles, MV-MERS; black diamonds, MV_{SCHW}. For statistical analysis of VNT data, Kruskal-Wallis test was performed in combination with Dunn's multiple comparisons test to compare all pair means.*, $P<0.05$ **, $P<0.01$.

Tiwarekar et al. Suppl. Figure S6 – T cells in mice three weeks after boost

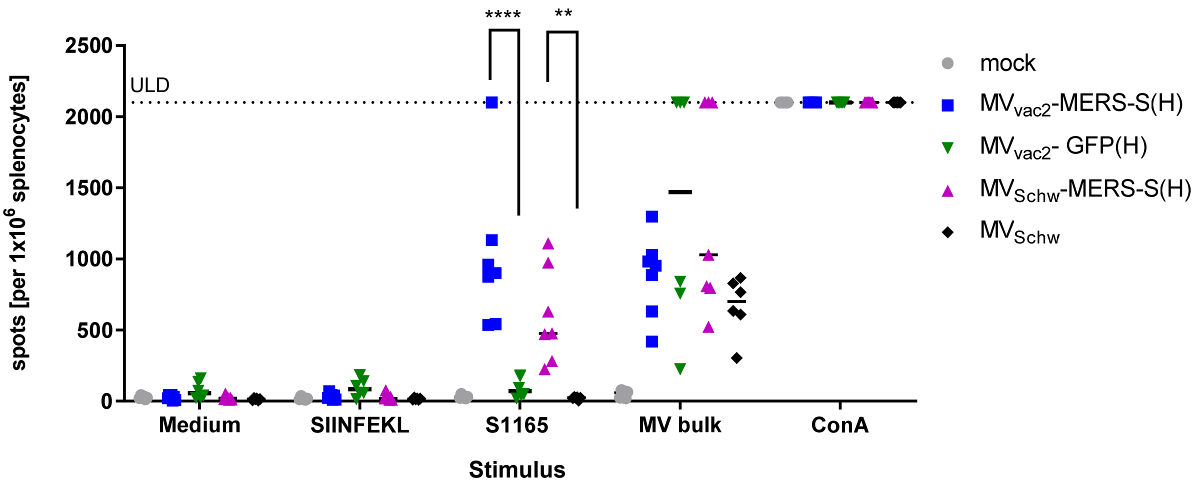

Suppl. Fig. S6: Reactivity of splenocytes after antigen-specific re-stimulation 3 weeks after vaccination. IFN-γ-ELISpot analysis using splenocytes of mice (also depicted in Fig. 3) vaccinated on days 0 and 28 with indicated vaccines, isolated 21 days after boost immunization and after stimulation with the immunodominant MERS-S peptide S1165 or the irrelevant control peptide SIINFEKL. To analyze cellular responses directed against MeV, splenocytes were stimulated with 10 μg/mL MeV bulk antigens or were left unstimulated as controls (medium). The reactivity of splenocytes was confirmed by Concanavalin A (ConA) treatment (10 μg/mL). The number of cells per 1×10^6 splenocytes represent the amount of cells expressing IFN-γ upon re-stimulation. Dots represent individual animals, horizontal bars the mean per group (n = 5 - 7). Spots counts above countability of the software were set to the upper detection limit (ULOD). For statistical analysis of grouped ELISpot data, two-way ANOVA analysis was applied with paired Tukey's Multi comparison test as post hoc test. **, p<0.01; ****, p<0.0001.

Tiwarekar et al. Suppl. Figure S7 – CD4 ICS responses

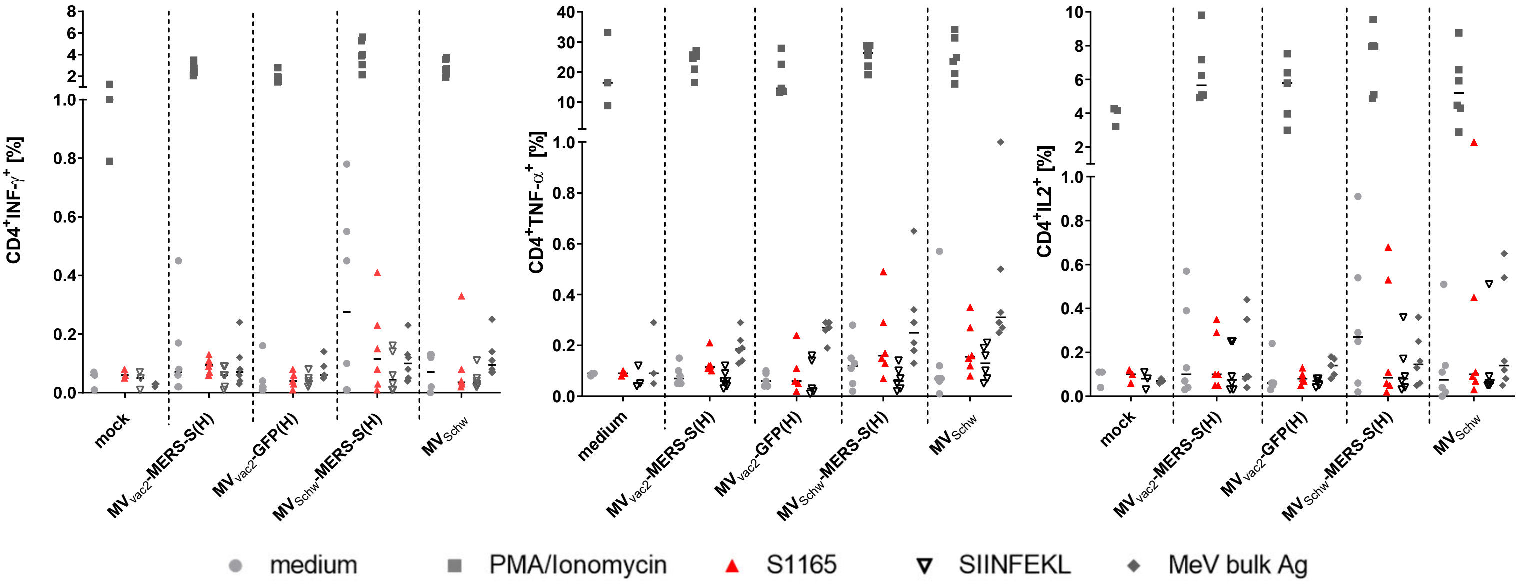

Suppl. Fig. S7: Reactivity of CD4⁺ T cells after antigen-specific re-stimulation. (**A**) Harvested splenocytes of vaccinated mice (same as depicted in Fig. 4) were re-stimulated with immunodominant MERS S peptide S1165 or irrelevant control peptide SIINFEKL. To analyze cellular responses directed against MeV, splenocytes were stimulated with 10 μg/mL MeV bulk antigens or were left unstimulated as controls (medium). The reactivity of splenocytes was confirmed by Concanavalin A (ConA) treatment (10 μg/mL). and subjected to intracellular staining (ICS) for IFN-γ, TNF-α, and IL-2, and stained for extracellular T-cell markers CD3 and CD4 for flow cytometry analysis. Quantification of flow cytometry data of cytokine-positive CD4⁺ T cells after incubation with indicated stimuli; reactivity of splenocytes was confirmed by Tetradecanoylphorbol-acetate and Ionomycin (TPA/Iono) treatment (10 μg/mL). Individual symbols represent individual animals, horizontal bars the median.

Tiwarekar et al. Suppl. Figure S8 – Suitability of IFNAR$^{-/-}$ mice for vaccination

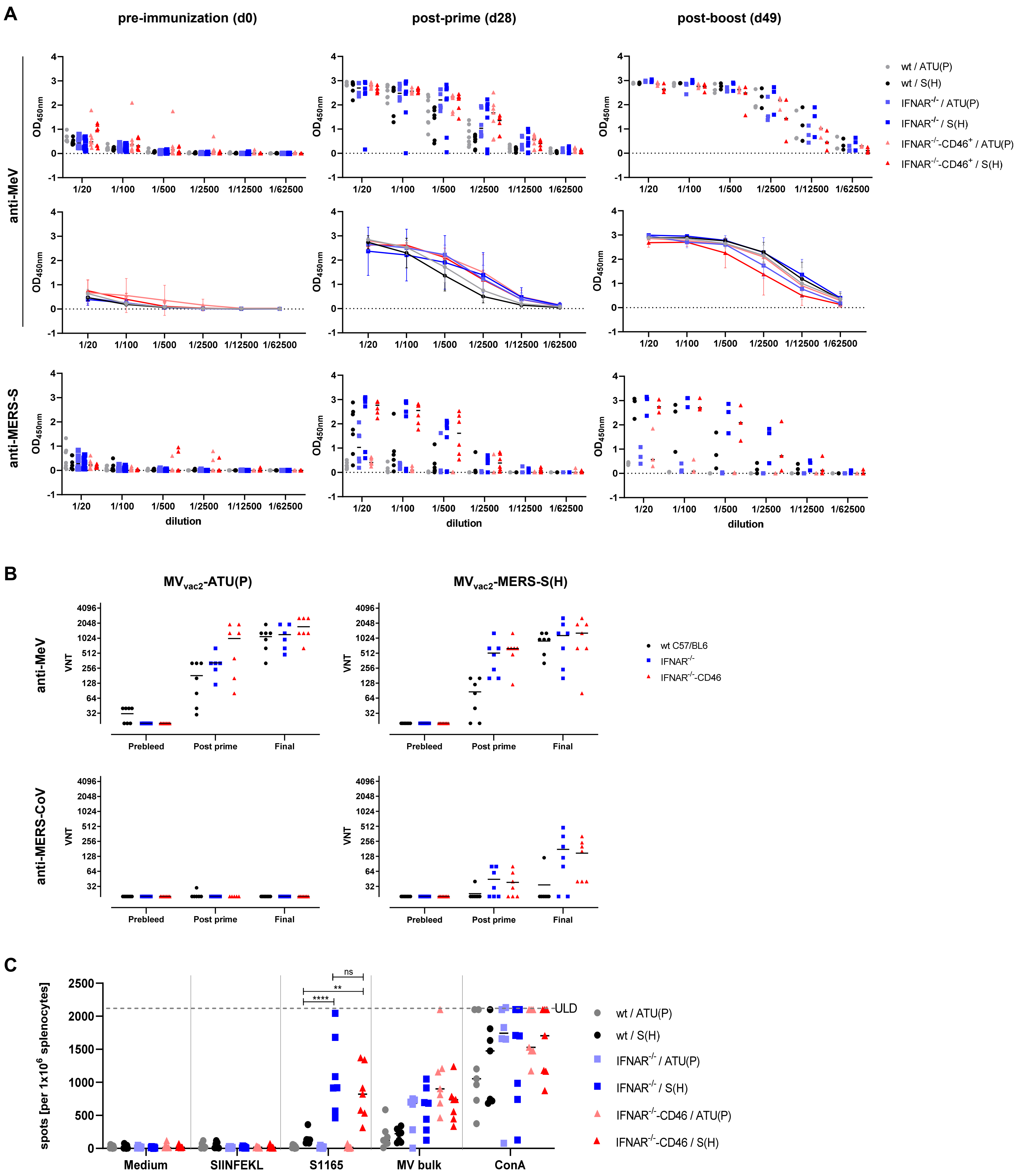

Suppl. Fig. S8: Immune responses in different mouse lines after vaccination with MeV-derived MERS vaccine candidates. To test the impact of the IFNAR knock-out or the hCD46 tg in $IFNAR^{-/-}$-CD46Ge mice, $IFNAR^{-/-}$-CD46Ge, $IFNAR^{-/-}$, or unmodified C57BL/6 mice (wt) were vaccinated on days 0 and 28 with MV_{vac2}-MERS-S(H) or vector control virus MV_{vac2}-ATU(P). Mice were sampled on day 0 (pre-immunization), day 28 after prime- (post-prime) and day 49 after boost-immunization when they were sacrificed and spleens were prepared for analysis of T cell responses (final). (**A, B**) Serum samples were analyzed for binding or neutralizing antibodies specific for MERS-CoV S or MeV. (**A**) Pan-IgG binding to recombinant MERS-CoV S (lower row) or MeV bulk antigens (top and middle row) were determined by ELISA via the specific OD_{450} value. Depicted are individual animals of each cohort (n = 6 - 7) (top and lower row), horizontal bars represent means or means with standard deviations (middle row). (**B**) Virus neutralizing titers (VNT) in vaccinated mice for MeV (upper row) or MERS-CoV (lower row) were calculated as reciprocal of the highest dilution abolishing infectivity. Dots represent single animals; horizontal line represents the mean per group. Y-axis starts at detection limit; all mice at detection limit had no detectable VNT. (**C**) IFN-γ-ELISpot analysis using splenocytes of the same mice isolated 21 days after boost immunization, and after stimulation with immunodominant MERS S peptide S1165 or irrelevant control peptide SIINFEKL. To analyze cellular responses directed against MeV, splenocytes were stimulated with 10 μg/mL MeV bulk antigens or were left unstimulated as controls (medium). The reactivity of splenocytes was confirmed by Concanavalin A (ConA) treatment (10 μg/mL). The number of cells per 1×10^6 splenocytes represent the amount of cells expressing IFN-γ upon re-stimulation. Dots represent individual animals, horizontal bars the median per group. Spots counts above countability of the software were set to the upper detection limit (ULD). For statistical analysis of grouped ELISpot data, two-way ANOVA analysis was applied with paired Tukey's Multi comparison test as post hoc test. **, p<0.01; ****, p<0.0001.

Tiwarekar et al. Suppl. Figure S9 – Establishing challenge model

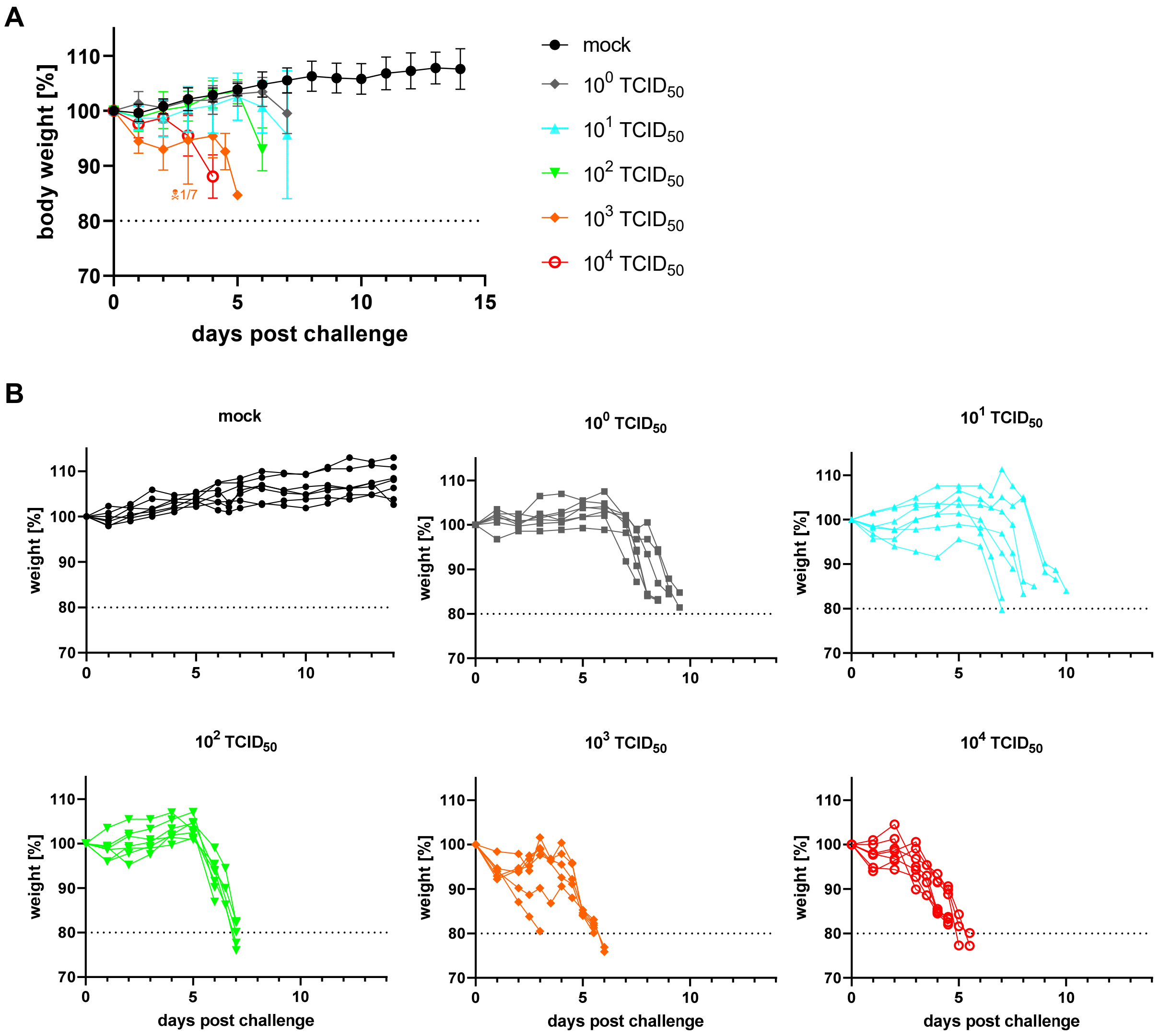

Suppl. Fig. S9. Weight of mice after challenge with different doses of MERS-CoV. (**A**) Weight loss in cohorts (n = 6 - 7) of IFNAR$^{-/-}$-hDPP4$^{+/-}$ mice infected as indicated – same animals as depicted in Fig. 5A are shown. Data points indicate means of each cohort, error bars the standard deviation. Curves end when first animals of the respective cohort were sacrificed (B) Weight of individual animals in indicated dose cohorts over time after infection.

Tiwarekar et al. Suppl. Figure S10 – Pre-challenge immunity

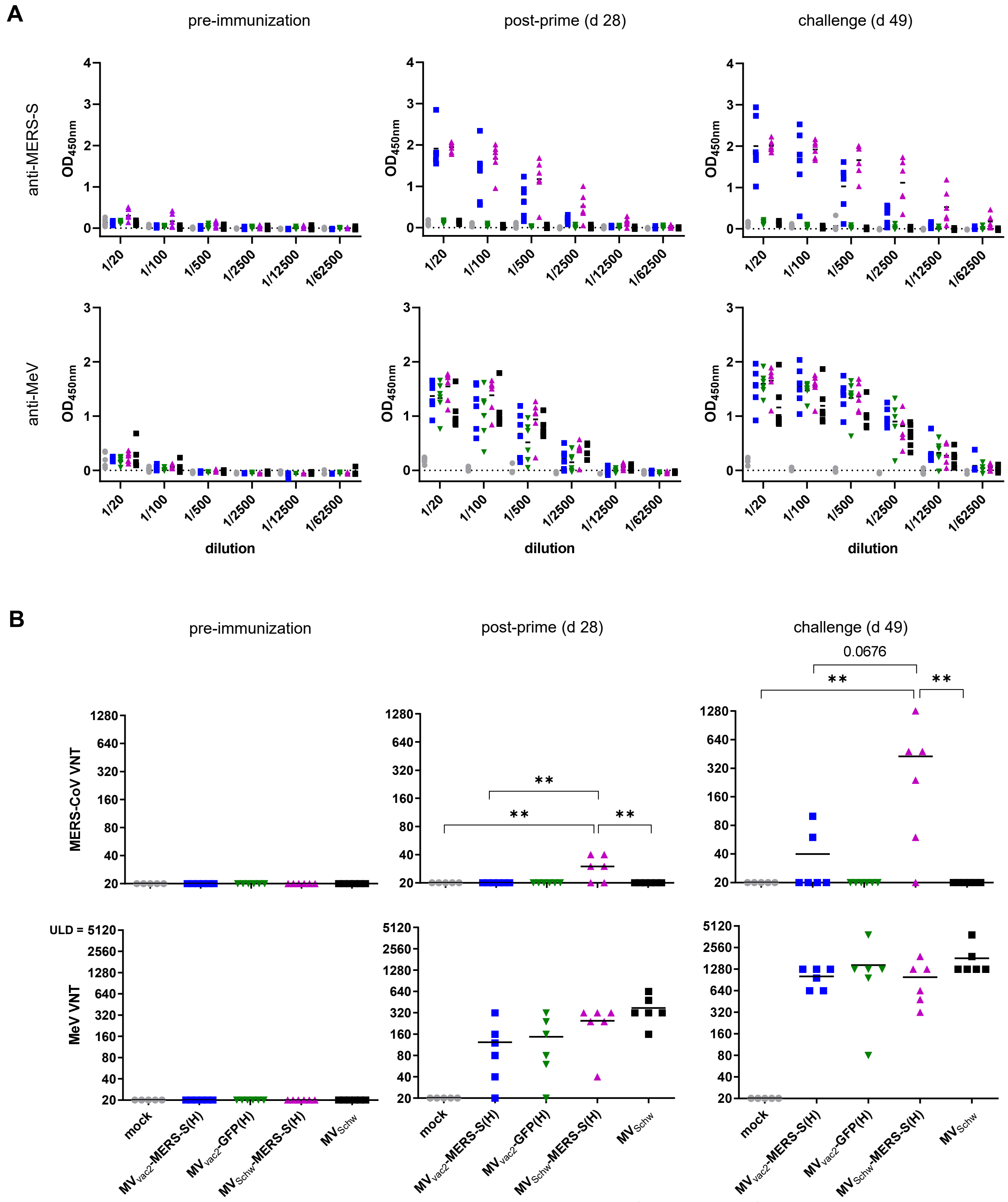

Suppl. Fig. S10: Immunity in vaccinate IFNAR$^{-/-}$-hDPP4$^{+/-}$ mice before challenge. Sera of mice (also depicted in Fig. 5 D-E) vaccinated on days 0 and 28 with indicated viruses were sampled on day 0 (pre-immunization), day 28 after prime- (post-prime) and day 49 after boost-immunization at the day of challenge infection (challenge) and analyzed for antibodies specific for MERS-CoV S or MeV. Medium-inoculated mice served as mock. (**A**) Pan-IgG binding to recombinant MERS-CoV S (upper row) or MeV bulk antigens (lower row) were determined by ELISA via the specific OD 450 nm value. Depicted are individual animals of each cohort (n = 6 - 7), horizontal bars represent means. (**B**) Virus neutralizing titers (VNT) in vaccinated mice for MERS-CoV (upper row) or MeV (lower row) were calculated as reciprocal of the highest dilution abolishing infectivity. Dots represent single animals; horizontal line represents mean per group. Y-axis starts at detection limit; all mice at detection limit had no detectable VNT. Grey circles, mock; blue squares, MV$_{vac2}$-MERS-S(H); green triangles, MV$_{vac2}$-GFP(H); magenta triangles, MV$_{SCHW}$-MERS-S(H); black diamonds, MV$_{SCHW}$. For statistical analysis of VNT data, Kruskal-Wallis test was performed in combination with Dunn's multiple comparisons test to compare all pair means. **,
